# Human Pluripotent Stem Cell-Derived Neural Cells as a Relevant Platform for Drug Screening in Alzheimer’s Disease

**DOI:** 10.3390/ijms21186867

**Published:** 2020-09-18

**Authors:** Juan Antonio Garcia-Leon, Laura Caceres-Palomo, Elisabeth Sanchez-Mejias, Marina Mejias-Ortega, Cristina Nuñez-Diaz, Juan Jose Fernandez-Valenzuela, Raquel Sanchez-Varo, Jose Carlos Davila, Javier Vitorica, Antonia Gutierrez

**Affiliations:** 1 Departamento Biologia Celular, Genetica y Fisiologia, Instituto de Investigacion Biomedica de Malaga-IBIMA, Facultad de Ciencias, Universidad de Malaga, 29071 Malaga, Spain; lauracaceres@uma.es (L.C.-P.); elisanchez@uma.es (E.S.-M.); marinamejias@uma.es (M.M.-O.); cristinand@uma.es (C.N.-D.); juanjofv@uma.es (J.J.F.-V.); raquelsv@uma.es (R.S.-V.); davila@uma.es (J.C.D.); 2 Centro de Investigacion Biomedica en Red sobre Enfermedades Neurodegenerativas (CIBERNED), 28031 Madrid, Spain; vitorica@us.es; 3 Departamento Bioquimica y Biologia Molecular, Facultad de Farmacia, Universidad de Sevilla, Instituto de Biomedicina de Sevilla (IBiS)-Hospital Universitario Virgen del Rocio/CSIC/Universidad de Sevilla, 41012 Sevilla, Spain

**Keywords:** human induced pluripotent stem cells (hiPSCs), disease modeling, Alzheimer’s disease, microglia, astrocytes, oligodendrocytes, 3D cultures, brain organoids

## Abstract

Extracellular amyloid-beta deposition and intraneuronal Tau-laden neurofibrillary tangles are prime features of Alzheimer’s disease (AD). The pathology of AD is very complex and still not fully understood, since different neural cell types are involved in the disease. Although neuronal function is clearly deteriorated in AD patients, recently, an increasing number of evidences have pointed towards glial cell dysfunction as one of the main causative phenomena implicated in AD pathogenesis. The complex disease pathology together with the lack of reliable disease models have precluded the development of effective therapies able to counteract disease progression. The discovery and implementation of human pluripotent stem cell technology represents an important opportunity in this field, as this system allows the generation of patient-derived cells to be used for disease modeling and therapeutic target identification and as a platform to be employed in drug discovery programs. In this review, we discuss the current studies using human pluripotent stem cells focused on AD, providing convincing evidences that this system is an excellent opportunity to advance in the comprehension of AD pathology, which will be translated to the development of the still missing effective therapies.

## 1. Introduction

Alzheimer’s disease (AD) is a highly debilitating and fatal neurodegenerative condition affecting the elderly population for which, to date, there is no known cure. AD accounts for more than two-thirds of dementia cases (currently 50 million worldwide and it will almost triple by 2050); thus, this brain disease is considered a major and increasing global health challenge [1]. The tissue proteopathic features of AD include the accumulation of aggregated amyloid-beta (Aβ) peptides and hyperphosphorylated Tau (p-Tau) leading to parenchymal and vascular amyloid plaques and to intraneuronal neurofibrillary tangles, respectively [2,3]. These protein lesions are accompanied by microglial and astroglial activation, dystrophic neurites, and synaptic damage followed by neuronal death. Most AD cases are sporadic and not linked to causative mutations; however, a significant number of genes associated with disease risk have been found (reviewed in [4]). Among these susceptibility genes, the *APOE4* variant and the recently described mutations in the triggering receptor expressed by myeloid cells 2 gene (*TREM2*) have a substantial impact on AD predisposition [5]. Less than 1% of AD cases are autosomal dominant familial AD caused by mutations in *APP*, *PSEN1*, or *PSEN2*, all involved in Aβ generation [6].

The amyloid cascade hypothesis considers the abnormal aggregation and accumulation of Aβ as the primary driving-force in AD pathogenesis [7]. However, all compounds targeting Aβ have failed so far throughout clinical trials [8], revealing a higher and still unsolved complexity for AD pathology. Over the last years, neuroinflammation has been posited as one of the main factors involved in AD pathology. In addition to astrogliosis (astroglia presenting a reactive state, widely reported as a feature present in AD brain; reviewed in [9]), microglia, which are resident innate immune cells in the brain, have acquired a relevant role in the disease [10]. Activated microglia accumulate around neuritic Aβ plaques, with many genes associated with AD risk being predominantly expressed in these cells (reviewed in [11,12,13,14,15,16]). Nonetheless, the precise role (detrimental or protective but with defective function) of these glial cells in AD pathogenesis is still not fully elucidated and may depend on disease stage and brain region. Emerging evidences support microglial dysfunction rather than an exacerbated neurotoxic microglial response in sporadic AD development [17].

The current knowledge on AD pathology has been built mostly by employing transgenic murine models overexpressing mutant human *APP*, *PSEN1*, or *PSEN2*, which have contributed to unraveling pathogenic pathways implicated in the disease [18]. Nonetheless, the poor translation of therapies developed in mouse models to AD patients reveals the lack of concordance between both systems, which have evidenced the urgent need for human-derived models to assure a better translation of biomedical research to the clinic. In this sense, the scarce access to human viable brain samples and the limited expansion of brain cells ex vivo have strongly limited the development of AD patient-derived models to study the disease. The advent of human-induced pluripotent stem cells (hiPSCs), which allows the reprogramming of a somatic cell (usually skin fibroblasts or blood leukocytes) towards a pluripotent stem cell (resembling embryonic stem cells) able to divide almost indefinitely and to give rise to almost any cell of the body, has opened a new paradigm in disease modeling, specially to neurodegenerative diseases (reviewed in [19]). The complexity of AD pathology, where most of the brain cell types are implicated, have precluded the development of studies aiming to unravel disease mechanisms employing hiPSCs. Nonetheless, over the last years, several studies have used hiPSC-derived neurons and glial cells for disease modeling, assessment of pathogenic pathways implicated in AD, and evaluation of candidate drugs in human-derived platforms in an effort to better assure the translation of discoveries towards the cure of AD.

In this review, we aim to compile and summarize the main studies reported to date employing hiPSCs from AD patients to generate neurons, glia, or 3D/organoid cultures composed of several brain cell types for AD modeling and/or drug screening efforts in an attempt to provide a better perspective of performed and ongoing investigations in this devastating disease.

## 2. HiPSC-Derived Neurons for AD Modeling and Platform for Screening

Neuronal dysfunction is the main phenomenon linked with the symptoms of AD. In AD patients, a prominent loss of synaptic connections occurs in almost all cortical areas examined, with synapse loss providing the best pathologic correlate of early cognitive decline [20]. As a consequence, there is a significant neuronal loss in AD patients, being more prominent in certain brain regions, directly related to those areas showing Tau pathology at different stages of the disease [21]. Moreover, neuronal vulnerability differs among different neuronal subpopulations, a phenomenon that is not always reproduced by the different animal models available [22].

### 2.1. Methods for the Generation of Neurons from hPSCs

Cortical neurons are the most relevant neuronal subtype for modeling AD. Protocols to generate this type of neurons from human pluripotent stem cells (hPSCs) have tried to reproduce in vitro the signals and cues occurring during in vivo neuronal development [23]. Initial approaches relied on the generation of 3D embryoid bodies from which, following a default differentiation program, neural lineage develops. Nonetheless, this approach led to poor purity, protracted differentiation, and variable results in terms of neural precursor cell (NPC) generation. Chambers and colleagues in 2009 demonstrated that double chemical inhibition of the SMAD (*Caenorhabditis elegans Sma* genes and the *Drosophila Mad*, Mothers against decapentaplegic) pathway converted PSCs into the neural lineage in 8–12 days with a high efficiency when culturing the cells in adherent conditions. They reported that further patterning of the cells with additional molecules and morphogens allowed for maturation of the NPCs generated into different neuronal subtypes [24]. This approach was adopted by most of the neuron-specific protocols developed afterwards.

In 2012, Shi and colleagues described a neuron-specific protocol for the generation of cortical neurons following similar temporal events during in vivo development. After neural specification by dual SMAD inhibition, their protocol relied on the purification and further expansion of NPCs. These NPCs were further cultured and allowed to mature for up to 100 days, recreating cortical neurogenesis and terminal neuronal maturation, with the generation of deep and upper layer cortical neurons in a specified temporal manner, similar to that in vivo. This methodology allowed to generate in vitro both excitatory and inhibitory cortical neurons resembling in vivo development [25]. In addition, knowledge about the specific signals that predominate in different brain region and are responsible for the generation and development of different neuronal subtypes has allowed the development of specific protocols for the derivation of specific neuronal subtypes as dopaminergic [26], glutamatergic, GABAergic, cholinergic, or motor neurons [27].

Despite their capacity to reproduce in vivo neurogenesis, dual SMAD inhibition-based protocols require long differentiation schedules to generate neuronal subpopulations with different degrees of maturity which, depending on the application, may represent a hurdle of the approach. By the beginning of the 2010s, few groups described the direct conversion of functional neurons from human fibroblasts, mediated by the overexpression of proneurogenic transcription factors (TFs) [28,29,30]. Based on this knowledge, the group of Südhof and Wernig described the directed generation of functional neurons from hPSCs in less than 2 weeks mediated by the exogenous overexpression of the TF *neurogenin 2* (*NGN2*). This approach allowed the generation of an almost 100% pure and homogeneous excitatory neuronal population from both human embryonic stem cells (hESCs) and hiPSCs which were electrophysiologically active and form synapses both in vitro and when transplanted into in vivo models [31]. In addition, this approach is valid for disease modeling and for assessing the deleterious mechanisms of teratogenic drugs [32]. Due to its robustness and efficiency, this approach has been widely used as a method to generate cortical neurons for disease modeling, as will be detailed below. Nonetheless, as it generates a homogeneous neuronal population, this approach might mask phenotypic differences within different neuronal subpopulations, as may be the case for AD.

**Figure 1:**
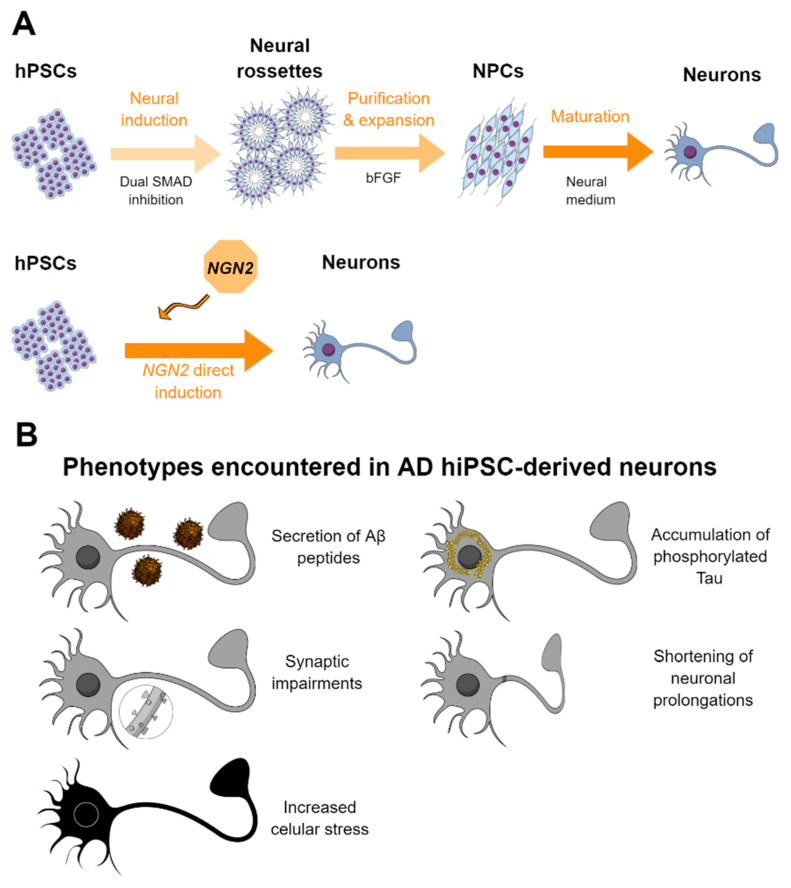
(**A**) The two most used approaches for the derivation of human pluripotent stem cell (hPSC)-derived neurons are represented: dual SMAD (*Caenorhabditis elegans Sma* genes and the *Drosophila Mad*, Mothers against decapentaplegic) inhibition-based protocols (above) and direct generation of neurons by the exogenous overexpression of *NGN2* (below). (**B**) The main phenotypes encountered in neurons derived from iPSCs of AD patients are presented. hPSCs: human pluripotent stem cells; iPSCs: induced pluripotent stem cells; bFGF: basic fibroblast growth factor; SMAD: *Caenorhabditis elegans Sma* genes and the *Drosophila Mad*, Mothers against decapentaplegic; *NGN2*: *neurogenin 2*; NPCs: neural precursor cells; Aβ: amyloid beta; AD: Alzheimer’s disease.

A summary of the most employed methodology for the derivation of hiPSC-derived neurons is represented in Figure 1A.

### 2.2. HPSC-Derived Neurons in AD and Drug Screening

As accumulation of Aβ is a major specific hallmark of AD, studies on AD have widely focused on the generation of hPSC-derived neurons from sporadic or familial cases with mutations in the genes associated with Aβ production (*APP*, *PSEN1*, or *PSEN2*) assessing whether aberrant accumulation of Aβ can be reproduced in vitro and assessing its functional consequences and/or suitability in the platform for drug assessment. In 2011, Yagi and colleagues first described the generation of induced pluripotent stem cells (iPSC)-derived neurons from familial AD (fAD) cases with mutations in *PSEN1* and *PSEN2* genes, finding that these cells presented higher Aβ_1–42_ production, which was reduced when cells were treated with specific gamma-secretase inhibitors, suggesting the potential of these cells to serve for identification and validation of candidate drugs [33]. A few months later, Israel and colleagues described the generation of iPSC-derived neurons from sporadic AD (sAD) and fAD patients with a duplication in the *APP* gene (*APP Dp*) as well as from healthy controls (HCs). Although they generated functional neurons from all subjects, they found that neurons derived from the two fADs and from one sAD case showed aberrant secretion of Aβ_1–40_. In addition, this was accompanied by higher levels of phosphorylated Tau protein and active glycogen synthase kinase-3b (aGSK-3b) and with accumulation of large RAB5^+^ endosomes, probably linked with the processing of APP-derived peptides. In addition, they demonstrated that this platform responded to drugs, as treating the generated neurons with beta-secretase inhibitors but not gamma-secretase inhibitors caused significant reductions in phospho-Tau and aGSK-3b levels, suggesting a direct relationship between APP proteolytic processing, but not Aβ, in GSK-3b activation and Tau phosphorylation in human neurons [34]. Muratore and colleagues generated iPSC-derived neurons from fAD patients with the London familial *APP V717I* mutation and found that, during in vitro maturation, cells notably increased their levels of APP and Aβ production, with an altered APP processing, leading to the secretion of Aβ_42_ and Aβ_38_ isoforms. Notably, this was accompanied with an increase in total and hyperphosphorylated Tau levels, which could be reversed using Aβ-blocking antibodies, therefore linking Aβ and Tau pathologies in iPSC-neurons [35]. Balez et al. reported that AD neurons showed a hyperexcitable calcium signaling phenotype, elevated levels of nitrite, increased cytotoxicity and apoptosis, reduced neurite length, and increased susceptibility to inflammatory stress, phenotypes that were mostly reversed by short-term treatment with apigenin (a plant polyphenol), suggesting that anti-inflammatory compounds may help in AD pathology [36].

Nonetheless, the studies described above were not able to reproduce the main pathogenic feature present in AD brains, that is synaptic loss. Nieweg et al. using HC-derived glutamatergic and GABAergic neurons found that exposing the cells to Aβ for several days led to a reduction of synapses and reduction of electrophysiological activity, without leading to cell death [37]. Similarly, Hu and colleagues derived neurons from subjects with *PSEN1* mutation, *APP* duplication, and chromosome 21 trisomy, and the secretome of generated neurons was injected into rat brains, finding that all of them caused synaptic dysfunction, resulting in inhibition of hippocampal long-term potentiation mediated by Aβ peptides or extracellular Tau. Notably, in all cases, synaptotoxicity was relieved by antibody blockade of the cellular prion protein, a sensor for protein misfolding [38].

Recently, Chang and colleagues derived neurons from fAD patients with *APP D678H* mutation and reported aberrant accumulation of intracellular and secreted Aβ_1–42_ and Aβ_1–40_ peptides, increased activation of GSK3β, hyperphosphorylation of Tau, impaired neurite outgrowth, downregulation of synaptophysin, and increased caspase 1 activity. Notably, these phenotypes were not present in an unaffected sibling. Treatment with the indole compound NC009-1 partially restored aberrant phenotypes, supporting the fact that iPSC-derived neurons can be employed for the assessment of candidate drugs [39]. Yang and colleagues generated *PSEN1* mutant AD-derived neurons and found, apart from higher levels of Aβ_42_ and Tau phosphorylation, an accelerated neuronal differentiation in mutant cells accompanied by a higher prevalence of apoptosis within the NPC population. Performing gain or loss of function experiments, they found that mutant variants of *PSEN1* were responsible for these pathogenic phenotypes [40]. Similarly, Arber and colleagues found an increased secretion of long Aβ peptides (Aβ_40_, Aβ_42_, and Aβ_43_) in neurons from fAD patients with *APP* and *PSEN1* mutations. They proposed that this phenomenon was caused in *APP V717I* mutants by alterations in the gamma-secretase cleavage site preference and in *PSEN1* mutants by reduced activity of the gamma-secretase. They demonstrated that iPSC-derived neurons can be employed for modeling and studying the mechanisms involved in Aβ production, as occurs in humans in vivo [41].

Apart from most phenotypes shown by neurons derived from AD patients with familial mutations, some other groups have described additional phenotypes which may have important roles in the disease. In this sense, Woodruff and colleagues found that gene-edited neurons carrying fAD mutations in *APP* or *PSEN1* genes had defects in the recycling state of endocytosis and soma-to-axon transcytosis of APP and lipoproteins. The authors suggested that the decreased lipoprotein endocytosis and transcytosis to the axon may represent a neuron-specific impairment in endocytic axonal delivery of lipoproteins, compromising synaptic maintenance in fAD. Notably, these defects in endocytosis were rescued by beta secretase inhibition [42]. In this line, Martín-Maestro and colleagues described alterations in the autophagic pathway in *PSEN1 A246E* fAD patient-derived neurons. This led to impairments in mitophagy (recycling of mitochondria), a pathway the authors also found altered in sporadic cases, suggesting that this is a common phenomenon in AD which can be studied with iPSC-derived neurons [43]. The same group described similar findings in NPCs from fAD patients with *PSEN1 M146L* mutation, which showed several mitochondrial respiratory chain defects together with aberrant mitophagy produced by autophagy induction blockage. Notably, treating these cells with the autophagy-stimulating drug bexarotene restored autophagy and compensated mitochondrial abnormalities [44]. Kwart and coauthors generated a large panel of fAD-derived and gene-edited iPSC lines with mutations in *APP* and *PSEN1* genes. At the transcriptome level, they found important gene expression changes among samples, with a common dysregulation of endosomal pathways in mutant cells. Importantly, endosomal dysfunction correlated with accumulation of C-terminal fragments produced by the processing of APP, instead of Aβ, and this could be rescued by pharmacological modulation of beta-secretase (BACE) [45].

Oxidative stress is another pathological hallmark present in AD brains, but its relationship with Tau or Aβ pathology is poorly understood. By using iPSC-derived neurons induced by *NGN2* overexpression from sAD patients, Birnbaum et al. described that neurons from sAD patients showed increased production of reactive oxygen species (ROS) and displayed higher levels of DNA damage, which did not correlate with Aβ or Tau phosphorylation, suggesting that increased ROS production may precede amyloid and Tau pathology in AD [46].

Some studies have reported the derivation of specific neuronal subpopulations with relevance for AD pathology to address specific AD-associated phenotypes in these cells. Accordingly, Ortiz-Virumbrales and colleagues generated basal forebrain cholinergic neurons from fAD *PSEN2 N141I* mutant patients, as they are one of the first cell types affected in AD. Apart from higher production of Aβ_42/40_, they observed diminished electrophysiological activity in these cells, phenotypes which were rescued after Clustered regularly interspaced short palindromic repeats (CRISPR)/CRISPR-associated protein 9 (Cas9)-mediated gene correction, corroborating the presence of the *PSEN2* mutation as the causative agent of altered neuronal function [47]. Similarly, Moreno et al. derived human basal forebrain cholinergic neurons from *PSEN2 N141I* fAD patients to shed light on the possible mechanisms that lead type 2 diabetes patients to cognitive impairment. They found that treatment of generated neurons with insulin led to a reduction in the Aβ_40_/Aβ_42_ ratio, and it also corrected the altered calcium flux present on diseased cells, addressing to some extent the neurological consequences of aberrant insulin homeostasis [48].

Some authors employed iPSC-derived neurons as a platform to identify and modulate drug targets for AD. Kondo and colleagues built a platform based on fAD-derived neurons (*PSEN1 G384A*) where they tested the effect of >1000 compounds on Aβ production. After validation steps and chemical structure modeling, they found six leading compounds that showed dose-dependent Aβ_42_ reduction, identifying a combination of three of them (bromocriptine, cromolyn, and topiramate) as the most potent anti-Aβ combinations validated in several fAD and sAD-derived neuronal cells [49]. Following a similar approach, Jin et al. generated a medium-throughput platform constituted by iPSC-derived neurons generated by *NGN2* overexpression. On these cells, they evaluated the effects of soluble Aβ-enriched AD brain extracts on neurite outgrowth, finding that these extracts were toxic to the cells in direct proportion to the Aβ content of the samples. The addition of three Aβ-specific blocking antibodies (1C22, 3D6, or 266) counteracted Aβ-mediated toxicity, suggesting that the platform could be employed for assessing drugs modulating Aβ availability [50].

All these studies evidence the suitability of iPSC-derived neurons for modeling Aβ generation and aggregation. Nonetheless, the development of culture systems able to retain and promote Aβ aggregation is mandatory for a proper reproduction of Aβ pathology, as occurs in vivo. The development and fine-tuning of these systems for a high-throughput assessment of drugs intervening in these mechanisms would, undoubtedly, lead to the discovery of drugs able to diminish Aβ pathology.

Apart from studies on cells derived from fAD cases, some authors have focused on describing aberrant phenotypes on neurons derived from sporadic AD cases, mostly from *APOE4* patients. Wang and colleagues described that *APOE4* neurons had higher levels of Aβ secretion and Tau phosphorylation non-linked to Aβ, together with specific degeneration of GABAergic neurons. They found that converting *APOE4* to *APOE3* by gene editing rescued the phenotypes encountered, similar to that when APOE4-expressing neurons are treated with a small-molecule structure corrector, suggesting that this could be a possible therapeutic approach for sAD [51]. Lin et al., using gene-corrected isogenic lines, reported that iPSC-derived neurons from *APOE4* donors showed a more mature phenotype with higher number of synapses, more abundant early endosomes, and higher secretion of Aβ_42_. Moreover, APOE4 astrocytes and microglia showed a reduced capacity of Aβ uptake, results which were validated in mixed 3D cultures, suggesting that the presence of APOE4 alters the functioning of neural cells at different levels [52]. An accelerated neuronal differentiation of *APOE4* iPSC-derived neurons has also been observed by Meyer and colleagues, who observed an impaired proliferative capacity of NPCs. They found that nuclear lamina alterations in *APOE4* neural cells reduced nuclear translocation of the transcriptional repressor REST, which is strongly implicated in the altered transcriptome and differentiation state of *APOE4* neurons [53].

AD is considered a secondary tauopathy, with Tau pathology being a constitutive hallmark of the disease, but mutations in the *MAPT* gene (which encodes for Tau) have not been linked to the disease. Several studies have generated iPSC-derived cells from individuals carrying different *MAPT* mutations to model Tau pathology in vitro. Fong and colleagues described the generation of neurons from a patient harboring the *A152T MAPT* mutation, which presented increased Tau fragmentation and phosphorylation, leading to neurodegeneration and axonal degeneration. Genetic correction of the mutation restored those phenotypes and homozygous introduction of the mutation exacerbated the phenotypes, demonstrating the causality of this mutation in the phenotypes encountered [54]. In frontotemporal dementia (FTD) patients harboring *N279K* and *V337M MAPT* mutations, Ehrlich et al. derived hiPSC-derived neurons and observed pronounced Tau pathology with increased fragmentation and phosphorylation, decreased neurite extension, and increased but reversible oxidative stress response to inhibition of mitochondrial respiration. Furthermore, FTD neurons showed an activation of the unfolded protein response and disease-associated gene expression profiles. Employing NSCs derived also from FTDP-17 patients with the *N279K MAPT* mutation, Wren and colleagues reported an increased expression of 4R Tau isoforms, increased cellular stress, and impaired endocytic trafficking, with some of those findings verified using autopsy brains [55]. The Spillantini group performed a more exhaustive characterization of hiPSC-derived neurons from patients with the *N279K* and *P301L MAPT* mutations. In diseased neurons, they found an increase in the 4R/3R ratio of Tau expression, an earlier electrophysiological maturation and an increase in Tau phosphorylation, with some of these phenotypes also present in the brains of patients with these mutations [56]. To gain insights into the mechanisms conducting neuronal degeneration in patients with tauopathies, Imamura and colleagues generated hiPSC-derived neurons with different Tau mutations and found that diseased neurons showed dysregulation of the augmentation of Ca^2+^ transients evoked by electrical stimulation, which led to the release of misfolded Tau and cell death. Chemical inhibition of Ca^2+^ influx attenuated misfolded Tau and cell death, suggesting that neuronal activity may regulate neurodegeneration in tauopathy, and this is susceptible to therapeutic modulation [57]. Recently, Nakamura and colleagues generated neurons from *R406W MAPT* patients and observed a notable Tau fragmentation and mislocation, which led axons to display morphological and functional abnormalities that could be rescued by microtubule stabilization, providing insights into the mechanisms leading to Tau-induced degeneration [58]. To generate a robust hiPSC-derived model for tauopathies, we introduced in a footprint-free manner three *MAPT* mutations (*N279K*, *P301L* and *E10+16*) into hiPSCs and differentiated the cells towards cortical neurons, finding that mutant neurons expressed higher levels of 4R Tau, higher phosphorylation and aggregation of Tau, an increased electrophysiological activity, deficiencies in neurite outgrowth, aberrant sequence of differentiation to cortical neurons, and a significant activation of stress response pathways. RNA sequencing confirmed stress activation, demonstrated a shift toward GABAergic identity, and an upregulation of neurodegenerative pathways. Therefore, this hiPSC-derived model could be employed to assess the effect of candidate compounds on different neurodegenerative phenotypes [59].

Different groups have developed iPSC-derived platforms to evaluate drugs able to counteract Tau pathology. Medda et al. developed an iPSC-derived neuron platform to evaluate candidate compounds able to reduce Tau aggregation in a high-throughput compatible manner [60]. Wang and colleagues generated *NGN2*-induced cortical neurons cultured in 384-well plates to evaluate the effect of a library of 1280 pharmacologically active compounds on reducing Tau expression by immunostaining. They found that three compounds (clonidine, moxonidine, and metaproterenol, the latter two being agonists of adrenergic receptors) reduced Tau content without cell toxicity, suggesting that activation of adrenergic signaling pathways could represent Tau-lowering therapeutic strategies in humans [61]. Recently, van der Kant et al. employed iPSC-derived neurons from fAD and sAD patients as a high-throughput platform to test >1600 compounds for their potency to inhibit p-Tau accumulation. From this screen, they found several drugs able to reduce p-Tau levels, with several of them involved in the cholesterol synthesis pathway. After exhaustive analysis of the cholesterol pathway, they identified cholesteryl esters (CE), the storage product of excess cholesterol, as upstream regulators of Tau early during AD development, with CE also regulating Aβ secretion via independent pathways. They found that drug-mediated allosteric activation of CYP46A1 lowers CE levels in neurons with low toxicity, reporting a therapeutic possibility for AD [62].

All these reports indicate that iPSC-derived neurons from AD patients can serve as a proper platform to gain insights into the mechanisms involved in AD pathology and that they could be used as well as a platform to discover and validate candidate drugs in a human context. Summary of the findings of the main studies reported using hiPSC-derived neurons from or related to AD patients is presented in Table 1. Moreover, graphical summary of the main pathological findings described in hiPSC-derived AD neurons is represented in Figure 1B.

## 3. HiPSC-Derived Astrocytes for AD Modeling and Platform for Screening

### 3.1. Definition and Functions of Astrocytes

Astrocytes are one of the most abundant cells present in the central nervous system (CNS) of mammals, being essential for brain development and homeostasis [67,68,69]. During CNS development, astrocytes are key in shaping the mature neural connections, as they actively engulf both excitatory and inhibitory synapses, a function that continues also in the adult [70] and pathological [71] brain. Astrocytes can modulate synaptic transmission and plasticity of excitatory synapses by the reuptake of glutamate from the synaptic cleft, helping circulation of this neurotransmitter and preventing excitotoxicity. In fact, most of glutamatergic synapses are monitored by a discrete astrocytic portion, with these communicating structures being known as tripartite synapsis, with a single astrocyte wrapping up to four neurons and hundreds of dendrites [72]. Astrocytes express two glutamate-specific transporters known as *GLT-1* (or excitatory amino acid transporter 2, EAAT2) and GLAST (or excitatory amino acid transporter 1, EAAT1), that control the glutamate availability at the synapse (reviewed in [73]). In addition to this, astrocytes are able to produce several molecules which act in synaptic modulation, with important effects on long-term potentiation and depression (LTP and LTD, respectively). Nonetheless, there is still important controversy on whether this phenomenon, known as gliotransmission, occurs under physiological conditions [74].

Astrocytes have an important role in metabolic support of neurons [75]. As a consequence of excitatory synaptic transmission, glutamate is delivered to the synaptic cleft, for which the excess is removed by astrocytes through their glutamate transporters, using energy from glycolysis for this process. As a consequence, lactate is produced by astrocytes, which is transported to surrounding neurons using lactate-specific transporters, a mechanism known as lactate shuttle [76]. Once inside the neuron, this lactate is used for obtaining energy through oxidative phosphorylation. Astrocytes also contribute to the blood–brain barrier by emitting perivascular endfeet, controlling water (astrocytes express aquaporins) and solute exchange between blood and brain parenchyma. This is key to synchronizing neuronal activity with blood flow, a process known as neurovascular coupling [77].

Apart from all these functions, astrocytes have an important role in inflammation and response to injury [68,78,79]. When a pathological insult occurs in the brain, they react to these changes becoming reactive, acquiring a hypertrophic phenotype with increased expression of glial fibrillary acid protein (GFAP) and altering and thickening their processes, a phenomenon known as astrogliosis [80]. Astrogliosis is considered a defense mechanism that controls inflammation and the blood–brain barrier integrity. An important contribution of astrogliosis is the isolation of non-injured tissue from damaged areas and the support of neuronal circuit and tissue regeneration after neurological insults [79].

After trauma, chronic infection, or other CNS diseases, a multicellular inflammatory response takes place where resident and infiltrating inflammatory cells have a key role in counteracting acute insults. These immune cells are able to elicit activation of astrocytes, leading to at least two different phenotypes based on transcriptome analysis named A1 (related with neuroinflammation) and A2 (related to ischemia) [81]. A1 astrocytes synthetize inflammatory cytokines and upregulate complement cascade genes, leading to the engulfment of synapses, therefore presenting, at first, detrimental functions. On the other hand, A2 astrocytes upregulate neurotrophic factors promoting cell survival, neuronal growth, and repair of synapses [82]. However, this A1/A2 binary classification may not be sufficient to explain the emerging phenotypic diversity of reactive astrocytes across brain disorders [83,84,85]. In fact, in response to injury, astrocytes seem to elicit differential responses depending on the insult they encounter, suggesting that astrocytic phenotype may respond to the microenvironment they encounter [86].

Apart from different astrocytic phenotypes in response to CNS insults, the diversity of astrocyte subpopulations within the homeostatic CNS has long been debated [83,87,88]. Based on morphology, several subtypes of astrocytes within the human brain have been described, with most of them classified as protoplasmic (present in gray matter) or fibrous (found in white matter) astrocytes, although additional astrocytic morphological subtypes as subpial interlaminar astrocytes or perivascular astrocytes are also present [89]. John Lin et al. selecting astrocytes by the expression of the pan astrocytic marker Aldh1L1, identified five different astrocytic subpopulations in the mouse brain attending to the positive or negative expressions of the extracellular markers CD51, CD63, and CD71. The proportions of these five subpopulations varied within the brain region examined, with each of these subpopulations differentially supporting synaptogenesis between neurons, and these astrocytic subpopulations seem to have analogous counterparts in the human brain [90]. Using single-cell RNA sequencing, five transcriptomically distinct astrocyte subtypes in adult mouse cortex and hippocampus have been described, for which the proportions vary among brain regions. Differences in their transcriptome point towards different specialized functions which still have to be revealed [91]. This astrocytic variability has been further emphasized employing the single-cell RNAseq technology, as the Rowitch group reported different astrocyte identities within the mouse cortex layers, which originated during development and remained through adulthood, following a pattern different to that of neurons [92]. These studies reveal a great astrocytic heterogeneity within the brain that may be the result of cell specializations to the surrounding microenvironment during development, homeostasis, or pathological insults. Further studies are needed to fully address and characterize the astrocytic diversity present in the human brain.

### 3.2. Astrocytes in AD

One of the main alterations found in the brains of AD patients is a strong astrocytic reactivity (astrogliosis) around plaques of amyloid beta, visualized as hypertrophic GFAP^+^ astrocytes polarized towards these protein accumulations [93], with the number of reactive astrocytes in the vicinity of Aβ plaques increasing with disease progression [94]. In relation to Tau pathology, astrocytes present a strong response to p-Tau in the initial stages of the disease (Braak I–II), which seems to vary with disease course, with astrocytes presenting a dysfunctional phenotype at advanced stages (Braak V–VI) [95]. Different studies suggest that both reactive and degenerative astrocytes coexist within the AD brain, depending on their location to Aβ plaques and Tau laden [96].

Despite the morphological changes suffered by astroglia in the context of AD having long been reported, little is known about the role that astrocytes play in AD pathogenesis (reviewed in [9]). In relation with Aβ plaques, reactive astrocytes limit the progression of Aβ plaques [97] likely through direct phagocytosis and degradation of Aβ species [98]. In line with this phagocytic capacity, we have recently shown that astrocytes are able to engulf and digest plaque-associated presynaptic dystrophic neurites in both amyloidogenic mouse models and AD brains, a phenomenon with little if any contribution from microglia [99].

Transcriptomic studies have reported that astrocytes in AD patients present an upregulation of genes involved in immune response and phagocytosis as well as significant changes in mitochondrial genes [100]. Similar studies performed in Aβ-enriched mouse models suggest that astrocytes acquire an inflammatory phenotype and downregulate the expression of genes involved in neuronal support and neuronal signaling, likely contributing to the neuronal dysfunction and cognitive decline present in AD [101]. In this sense, using human samples and single-nucleus RNA-sequencing, Colonna´s group recently reported the association between AD and the downregulation of genes implicated in the metabolic coordination between astrocytes and neurons [102].

Recent single-cell RNAseq studies have provided additional information about the intrinsic features present in AD astrocytes. Mathys et al. reported that, in contrast to neurons and similarly to the other glial cells, astrocytes present a general upregulation of expression markers, with *GFAP*, *SYTL4*, *MT1E*, *ZFP36L1*, *MT2A*, and *GJA1* standing out, but a downregulation of *APOE*. Among astrocytes, they found a specific subpopulation which was differentially associated to AD and characterized by the expression of *GLUL* and of the AD risk factor *CLU* [103]. Using similar approaches analyzing entorhinal cortex from AD and HCs, Grubman et al. described the presence of specific astrocytic subpopulations in AD patients. Notably, they described that the *TFEB* gene, which was upregulated in diseased astrocytes, acted upstream of ten AD genome-wide association studies (GWAS) loci (*BIN1*, *CLDN11*, *POLN*, *STK32B*, *EDIL3*, *AKAP12*, *HECW1*, *WDR5*, *LEMD2*, and *DLC1*), which are also dysregulated in AD astrocytes. They propose that this upregulated regulatory module underlies the cellular transitions from control to AD states in specific astrocyte subpopulations [104].

Recently, the presence of a disease-associated astrocyte (DAA) subpopulation has been described in an AD mouse model (5×FAD), associated also with brain aging [105]. These DAAs emerge in AD models before pathological manifestations and are characterized by presenting the expression of a unique set of genes, including genes involved in endocytosis, complement cascade, and aging. Interestingly, the authors also found an analogous astrocytic population in aged AD patient brains. Notably, these DAAs shared several characteristic genes with the recently described disease-associated microglia population [106,107], including the AD risk gene *Apoe* and the *Ctsb*, *Ctsd*, and *Ctsl* genes, encoding proteins (cathepsins B, D, and L) implicated in AD pathogenesis [105]. Using spatial transcriptomics and in situ sequencing, recently, the De Strooper lab identified several Aβ plaques-induced genes that are mostly produced in coordination by microglia and astrocytes, suggesting the presence of an astrocytic population responding to AD insults [108].

In summary, these studies suggest that astrocytes are very implicated in AD pathology. Nonetheless, further studies are required to elucidate the precise role that reactive astrocytes have on AD pathogenesis.

### 3.3. Methods for the Derivation of Astrocytes from hPSCs

As specified above, mouse models have been widely used for AD modeling, but murine astrocytes have important divergences with human counterparts [109], which make them not the best platform to be used for disease modeling and drug screening. Phenotypically, hominid astrocytes are more than two times larger than murine counterparts and their degree of complexity is much higher, as they emit up to ten-fold more processes. Functionally, their intracellular calcium activity is faster and more prolongated that the one present on murine astrocytes. These features together with a higher variability of astrocyte subtypes in the human neocortex indicate key intrinsic differences between human and murine astrocytes [110]. At the transcriptome level, murine and human astrocytes present important differences, which are directly related to their different functionalities [111]. Therefore, it is tentative to state that human-derived astrocytes must be used to address the role that these cells play on AD pathology.

Since the implementation of hESC and hiPSC technologies, several groups have developed specific protocols for the generation of astrocytes from hPSCs which mimic the in vivo ontogeny of these cells [112,113,114,115]. It is accepted that the developmental origin of astrocytes is similar to that of neurons in terms of regional specification, although it occurs later, during the late embryonic stage and early postnatal period. Astrocyte precursors are specified from neural precursor cells via Notch signaling [116], and then, these precursors migrate away from the germinal zones in a radial manner to specific regions of the brain, where they differentiate and mature, forming region-specific astrocytic subpopulations [117].

Therefore, protocols for the generation of hPSC-derived astrocytes generally consist of four main stages: (1) specification of PSCs towards neuroepithelial cells (through the formation of an embryoid body in 3D or in attached cultures by means of a chemical double inhibition of SMAD signaling [24]); (2) induction and expansion of an homogeneous neural stem cell population culturing the cells in neural medium with the presence of specific morphogens as FGF8, retinoic acid, or sonic hedgehog signaling; (3) specification towards glial lineage and expansion in the presence of epidermal growth factor (EGF) and FGF2; and (4) astrocytic maturation culturing the cells in members of the interleukin 6 family as leukemia inhibitory factor (LIF) or ciliary neurotrophic factor (CNTF), which activate the Janus kinases-signal transducer and activator of transcription proteins (JAK-STAT) pathway, key in astrocytic specification [117]. During the maturation step, most of these protocols employ serum, an undefined component of the culture medium which irreversibly primes maturating astrocytes towards a reactive state different from homeostatic astrocytes [111]. According to this, serum-deprived protocols for the generation of astrocytes have been developed to generate homeostatic astrocytes [113,118].

**Figure 2:**
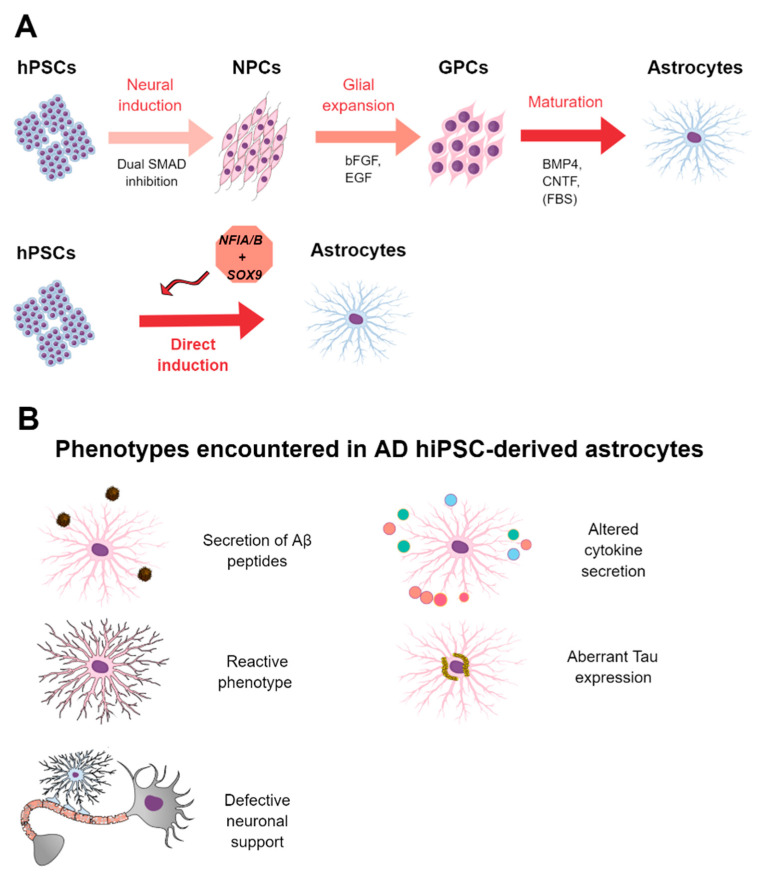
(**A**) The two most used approaches for the derivation of hPSC-derived astrocytes are represented: dual SMAD inhibition-based protocols and expansion of glial precursor cells (GPCs) (above) and direct generation of astrocytes by the exogenous overexpression of *NFIA/B* plus *SOX9* (below). (**B**) The main phenotypes encountered in astrocytes derived from iPSCs of AD patients are presented. hPSCs: human pluripotent stem cells; iPSCs: induced pluripotent stem cells; bFGF: basic fibroblast growth factor; EGF: epidermal growth factor; BMP4: Bone morphogenetic protein 4; CNTF: Ciliary Neurotrophic Factor; FBS: Fetal Bovine Serum; NPCs: neural precursor cells; GPCs: glial precursor cells; Aβ: amyloid beta; AD: Alzheimer’s disease.

Protocols developed by mimicking in vivo astrocytic development require long culture procedures (3–6 months from the PSC stage) to obtain mature astrocytes. Based on this and similar to that described for neurons, transcription factor (TF)-mediated direct generation of functional astrocytes has been performed by few groups. Canals and colleagues were the first to describe direct generation of mature astrocytes from hPSCs by the forced expression of the TFs *SOX9* and *NFIB* in just 2–3 weeks. They demonstrated that generated astrocytes resembled mature primary astrocytes, were functional both in vitro and when transplanted into immunodeficient mice, and served to model neurodegenerative diseases [119]. Similarly, Li et al. reported that forced expression of *NFIA* and *SOX9* sped up the generation of astrocytes. Moreover, they observed that combination of this approach with the addition of specific morphogens led to the derivation of different brain region-specific astrocytes [120]. In addition, Tchieu and colleagues reported that transient expression of *NFIA* led to a gliogenic switch of the differentiating neural population, leading to an accelerated generation of functional astrocytes [121]. On the other hand and in an attempt to generate astrocytes with a level of maturity comparable with adult astrocytes, Sloan and colleagues described the generation of hPSC-derived astrocytes in 3D spheroids cultured for up to 20 months, after which they generated astroglial cells similar to adult astrocytes and, therefore, could be best employed for disease modeling purposes [122]. A summary of the methodology to generate hiPSC-derived astrocytes is depicted in Figure 2A.

### 3.4. HPSC-Derived Astrocytes in AD and Drug Screening

Although astrogliosis present in AD brains is a phenomenon known from early descriptions of the disease, still, the precise role of astrocytes in AD is undetermined [9]. This has precluded the study of AD patient-derived astrocytes to unravel if these cells present intrinsic AD deficiencies, with few studies performed in the past few years addressing this issue. In 2013, Kondo and colleagues described the generation of iPSC-derived neurons and astrocytes from sAD and fAD patients with *APP E693*Δ mutation. Although it was more prominent in iPSC-derived neurons, generated astrocytes from both sAD and fAD patients accumulated Aβ oligomers intracellularly. This was accompanied by an upregulation of the endoplasmic reticulum (ER) and reactive oxidative response (ROS) stress pathways which, apparently, did not alter their capacity of glutamate uptake. Notably, ER and ROS stress were resolved after treating the cells with a beta secretase inhibitor IV (BSI), which reduced the content of intracellular Aβ oligomers, indicating that these Aβ oligomers were the inductors of both ER and ROS stress. In addition to BSI, the authors tested three other drugs previously reported to improve ER and ROS stress (docosahexaenoic acid (DHA), dibenzoylmethane (DBM14-26), and NSC23766), finding that DHA alleviated stress induced by Aβ oligomers accumulation. This indicated that iPSC-derived astrocytes and neurons responded to compounds and could be used for evaluating the efficacy of drugs on AD patients from different disease subgroups [123].

Oksanen and colleagues, in 2017, generated iPSC-derived astrocytes from fAD patients with the *PSEN1* Δ*E9* mutation, a condition linked to an increase in the accumulation of Aβ [124]. Patient-derived astrocytes, but not those derived from isogenic corrected lines or healthy controls, presented alterations in Aβ metabolism since, although diseased astrocytes produced similar levels of total Aβ, *PSEN1*-mutant astrocytes increased 5-fold Aβ_1–42_ peptide secretion and showed reduced capacity of Aβ internalization and degradation, suggesting that *PSEN1*-mutant astrocytes could directly contribute to enhance Aβ load in AD brains. On the other hand, mutant astrocytes presented an altered cytokine secretion after stimulation with pro-inflammatory stimuli. Most importantly, this study described that *PSEN1*-mutant astrocytes may fail to provide proper support to neurons as they produced higher levels of reactive oxygen species and lower levels of lactate. Moreover, they presented and induced reduced calcium metabolism in neurons, most probably leading to dysfunctional neurons [124]. Jones and colleagues in 2017 described the generation of iPSC-derived astrocytes using fully defined medium from the NPC stage. They generated astrocytes from a patient with fAD (*PSEN1 M146L* mutation), from an *APOE4*^+/+^ patient with sporadic form, and from healthy controls. Although at the NPC stage they did not found differences between the subjects, astrocyte-specific defects were seen in both sAD and fAD-derived astrocytes. They found that both sAD and fAD-derived astrocytes presented an altered morphology, with much less cell complexity than healthy astrocytes, which redounded in significant reduction of both cell volume and area covered by diseased astrocytes. Moreover, they saw an aberrant location of canonical astrocytic markers as S100b, EAAT1, and GS, as astrocytes from both sAD and fAD patients showed accumulation of these proteins in the nuclei. Finally, researchers found an altered basal inflammatory cytokine production in diseased astrocytes. The authors suggested that these phenotypes were consistent with the presence of astrocytic atrophy in these patients, hampering astrocytic functionality and, most likely, contributing to disease pathogenesis [125].

In an attempt to generate a human-derived model for addressing the role that APOE play on AD, Zhao and colleagues generated iPSC-derived neurons and astrocytes from healthy *APOE3/3* and *APOE4/4* individuals. They observed that, during astrocytic maturation, they progressively increased the expression of APOE, with the APOE derived from *APOE4/4* subjects presenting lower lipidation status with significantly lower levels of cholesterol. To address the functional consequences of this differential status between APOE proteins, researchers identified that astrocytes derived from *APOE4/4* individuals had impaired supportive effects on neuronal viability and synaptic function. The authors posted this approach as a suitable platform to evaluate the mechanisms underlying APOE-related contribution to AD development and for the testing of candidate drugs [126].

Although not derived from AD patients but from patients with frontotemporal dementia (FTD) with mutations in Tau, Hallmann et al. in 2017 described the generation of iPSC-derived neurons and astrocytes from one FTD patient with the *MAPT* mutation *N279K* and from isogenic corrected lines and healthy controls. They found that both neurons and astrocytes displayed disease-specific phenotypes. Specifically, FTD-derived astrocytes showed aberrant expression of 4R Tau and occupied larger volumes compared to wild type cells. In addition, mutant astrocytes presented altered gene expression profiles, consistent with a higher cell reactivity, and upregulation in oxidative stress and unfolded protein response (UPR) pathways, detrimental responses which were able to be induced in iPSC-derived neurons when both cell types were co-cultured, suggesting a defective neuronal support by diseased astrocytes [127].

Although informative, the current number of studies employing iPSC-derived astrocytes from AD patients is reduced. Additional studies in this line are essential to address the role that astrocytes play in AD. A summary of the findings of the main studies reported using hiPSC-derived astrocytes from AD patients is present in Table 2. Moreover, graphical summary of the main pathological findings described in hiPSC-derived AD astrocytes is represented in Figure 2B.

## 4. HiPSC-Derived Oligodendrocytes for AD Modeling and Platform for Screening

### 4.1. Definition and Functions of Oligodendrocytes

Oligodendrocytes (OLs) are highly specialized cells of the CNS. They produce myelin, a multilayered and lipid-rich sheath that covers and insulates neuronal axons, allowing for proper and fast conduction of electrical signals along neuronal axons and providing metabolic support to them as well. OLs are the focus of many investigations as defects in the production or maintenance of this myelin sheet are pathological features of several neurological diseases including multiple sclerosis (MS) and leukodystrophies [128,129]. In addition to these diseases, recent investigations have revealed that defects in OL functioning contribute to the pathology of other brain diseases as amyotrophic lateral sclerosis (ALS), Huntington’s disease, or schizophrenia [130,131,132,133].

### 4.2. Oligodendrocytes in AD

Little knowledge is available about the role that OLs may play in AD pathogenesis. White matter abnormalities have long been reported in AD brains, with Aβ and Tau pathological hallmarks hypothesized to influence myelin integrity and OL activity [134], which could be influenced by *APOE* as well [135]. Loss of myelin integrity before Aβ and Tau pathologies has been reported in AD models, and it has been suggested that Aβ accumulation induces cell toxicity and apoptosis of OLs, which was partially reversed after Aβ_1-42_-specific blocking [136].

Using single-cell RNA sequencing of prefrontal cortex brain samples from HCs and AD patients with different pathological states, the Tsai group revealed that, within all cell types and altered pathways, myelination-related processes were recurrently perturbed in multiple cell types, suggesting that myelination has a key role in Alzheimer’s disease pathophysiology [103]. Similarly, Grubman et al., using single-cell sequencing of entorhinal cortex of AD patients, reported that, out of all major brain cell types, OLs have the greatest interindividual differences between AD patients, further implicating myelination in AD pathogenesis [104]. Using spatial transcriptomics and in situ sequencing, recently, the De Strooper lab identified several Aβ plaques-induced genes, with most of them produced by microglia and astrocytes. Strikingly, they also reported that the expression of oligodendrocytic genes increases in the vicinity of Aβ plaques at the beginning of the pathology and dramatically decreases at advanced stages, responses that vary within different brain regions, partly because differences in the Aβ load among them. In addition, they reported an oligodendrocyte Aβ-reactive state, suggesting that, together with activated astrocytes and microglia, OLs are clearly part of the multicellular inflammatory environment linked to Aβ plaques [108]. In line with this, studies performed in other neurodegenerative diseases have reported that OLs under pathological circumstances may develop inflammatory-related activities [137]. These evidences suggest that OLs may play an important role also in the inflammatory processes that occur in AD pathology, but we are just starting to understand the involvement of OLs in these processes and we are far from a full understanding about the role that OLs play on AD pathology.

### 4.3. Methods for the Derivation of Oligodendrocytes from hPSCs

**Figure 3:**
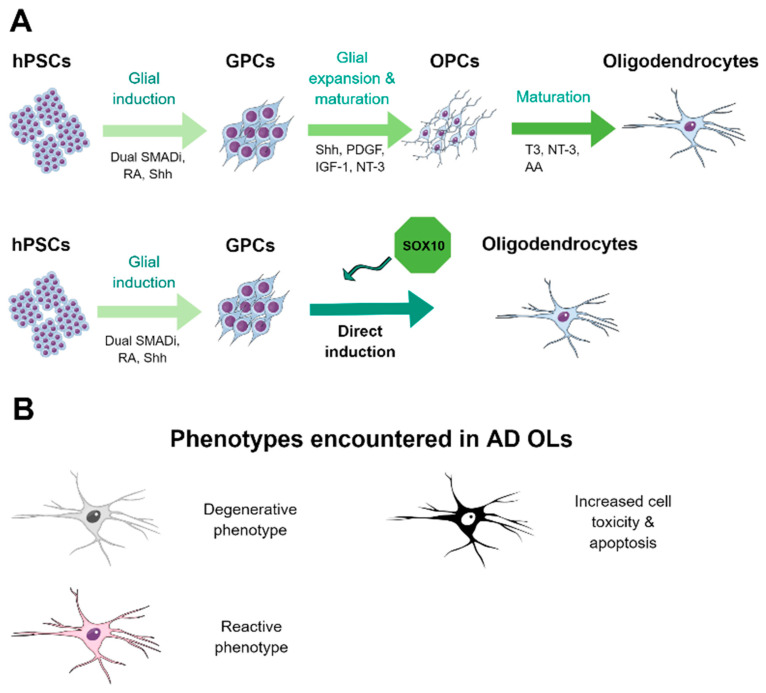
(**A**) The two most used approaches for the derivation of hPSC-derived oligodendrocytes (OLs) are represented: glial induction by dual SMAD inhibition in the presence of specific morphogens such as retinoic acid (RA) and sonic hedgehog (Shh); expansion of the oligodendrocyte precursor cells (OPCs) in the presence of platelet-derived growth factor (PDGF) and insulin growth factor 1 (IGF-1); terminal differentiation by the withdrawal of mitogens and addition of T3, NT-3, and ascorbic acid (above); and direct generation of OLs by the exogenous overexpression of *SOX10* on glial precursor cells (GPCs) (below). (**B**) The main phenotypes present in OLs from AD patients and models are presented. hPSCs: human pluripotent stem cells; iPSCs: induced pluripotent stem cells; RA: retinoic acid; Shh: sonic hedgehog; PDGF: platelet-derived growth factor; IGF-1: insulin growth factor 1; NT-3: neurotrophin 3; T3: triiodothyronine; AA: ascorbic acid; OPCs: oligodendrocyte precursor cells; GPCs: glial precursor cells; OLs: oligodendrocytes; AD: Alzheimer’s disease.

OLs emerge during late gliogenesis, which could be related with the fact that, from hPSCs, they only appear in cultures after several months of neural differentiation and with relatively low numbers [138]. Nonetheless, specific protocols for the generation of OLs from hPSCs have been developed [139,140]. These protocols rely on a neural induction phase followed by glial specification in the presence of specific morphogens such as retinoic acid and sonic hedgehog signaling; expansion of the oligodendrocyte progenitor population in the presence of platelet-derived growth factor (PDGF) and insulin growth factor 1 (IGF-1); and terminal differentiation by the withdrawal of mitogens and addition of triiodothyronine (T3), neurotrophin 3 (NT-3), and ascorbic acid [139,140]. Nonetheless, those approaches remain inefficient, as they represent long differentiation schedules and with low yield of mature OLs, hampering the employment of this technology for disease modeling. Recently, we and others have developed TF-based generation of mature OLs in just 20 days from the PSC stage by the exogenous overexpression of *SOX10* combined or not with other TFs [141,142]. This methodology allows for fast and efficient generation of hPSC-derived OLs that could be employed not only for disease modeling but also as a platform to assess the effect of candidate drugs involved in myelination in a high-throughput manner [141]. A summary of the methodology to generate hiPSC-derived OLs is depicted in Figure 3A.

### 4.4. HPSC-Derived Oligodendrocytes in AD

The little knowledge about the role that OLs play in AD has hampered the development of studies to characterize the oligodendroglial population derived from hiPSCs of AD patients. Only Ehrlich et al. reported that OLs derived from hiPSCs with *MAPT N279K* mutation had a higher susceptibility to oxidative stress-induced cell death [142]. Therefore, additional studies are needed to address the precise role that OLs play in AD pathology, which have attracted, to date, little attention.

## 5. HiPSC-Derived Microglia for AD Modeling and Platform for Screening

### 5.1. Definition and Functions of Microglia

Microglia are the innate immune cells of the brain. They have an important role in brain development, homeostasis, and neurological diseases which has been recently revealed, gaining an important relevance in current studies [143,144,145,146,147]. In contrast to the other brain parenchyma cells such as neurons or the other glial cells (astrocytes and oligodendrocytes), microglia do not have an ectodermal origin but, instead, they originate from yolk sack-derived myeloid progenitors during primitive hematopoiesis, which migrate and colonize the brain, developing branched processes and giving rise to microglia which remain during adulthood [148,149].

Since the discovery of microglia at the beginning of the 20th century by Pío Del Río-Ortega [150], extensive debate has taken place not only about the developmental origin of microglia but also on whether microglia populations are maintained during development and adulthood by peripheral macrophages as occurs in most tissues. Only recent studies employing lineage-tracing experiments and single-cell RNAseq have demonstrated that microglia only derive from brain resident counterparts without peripheral blood monocyte contribution (at least under homeostatic conditions) and that microglia populations are maintained locally [151,152].

Microglia examine the brain parenchyma, respond to damage, and maintain brain homeostasis, and interestingly, they differ in their abundancy, morpho-molecular signatures, and function across different brain regions [153]. However, under pathological conditions, these cells change to an activated state: they modify their morphology (larger cell body and less branched processes), proliferate, and secrete inflammatory factors. If the response to the pathological stimulus is not resolved, microglia undergo chronic activation, which could lead to deleterious consequences [17].

### 5.2. Microglia in AD

Even if neuroinflammation is a key component of AD pathology [10], the role that microglia play in AD has started to be elucidated in the last few years. In AD brains, activated microglia accumulate around Aβ plaques, suggesting that they may have an important role in Aβ phagocytosis and in plaques compaction. On the other hand, this leads to microglial activation, which releases proinflammatory cytokines, leading to neurotoxicity and Tau pathology exacerbation. Therefore, there are conflicting reports as it seems that microglia response in AD may play a dual role, being either beneficial or harmful [12,13], although this fact most probably will respond to differences in models used, brain regions, and/or disease stage examined. Our group has recently identified a degenerative/pathological status of microglia in the hippocampus of AD patients [154], supporting that a microglial dysfunctional phenotype underlies AD progression.

Transcriptomic studies have reported that gene pathways associated with immunity and microglia are upregulated in AD patients, with the microglia-specific gene *TYROBP* (coding for the adaptor protein DAP12) exerting a “hub” role within pathogenic pathways [155]. In addition, genome-wide association studies (GWAS) have revealed that polymorphisms in microglia-specific genes are associated with the risk of having AD. Among those polymorphisms, the *R47H* variant of the *TREM2* gene is the most firmly associated to sporadic AD, increasing 3–4 fold the risk of developing AD [5]. *TREM2* is a microglial receptor which plays important roles in microglial phagocytosis of apoptotic neurons, damaged myelin, and amyloid plaques [156]. It is now accepted that the *TREM2 R47H* variant leads to *TREM2* loss of function [157], but the functional consequences of this deficiency can be poorly assessed using current animal models [158]. Therefore, it is mandatory to develop human-derived models to assess not only the role that *TREM2* plays in AD but also the contribution of microglia to AD pathology.

### 5.3. Derivation of Microglia from hPSCs and their Use for Disease Modeling and Drug Screening

**Figure 4:**
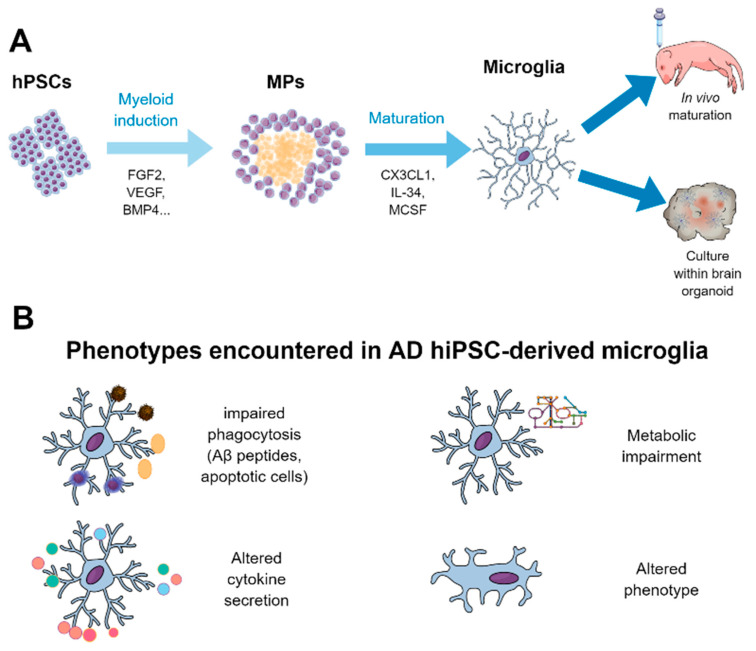
The most used protocol for the generation of microglia from hPSCs (**A**) and the main phenotypes encountered in microglia derived from iPSCs of AD patients (**B**). hPSCs: human pluripotent stem cells; iPSCs: induced pluripotent stem cells; bFGF: basic fibroblast growth factor; BMP4: Bone morphogenetic protein 4; VEGF: Vascular Endothelial Growth Factor; MCSF: Macrophage Colony Stimulating Factor; IL-34: interleukin 34; MPs: myeloid precursor cells; Aβ: amyloid beta; AD: Alzheimer’s disease.

The obtention of human primary microglia is very limited not only due to scarce tissue access but also due to reduced proliferation of these cells ex vivo. To overcome the latter, human immortalized cell lines have been generated, but with poor preservation of microglial features [159], evidencing the need to generate human microglia from virtually non-exhaustive sources such as hiPSCs. The delay in revealing the ontogeny of microglia has resulted in only recent developments of specific protocols to generate hiPSC-derived microglia. Previously, some protocols have tried to generate microglia from human monocytes [160] or not precisely reproducing microglial ontogeny [161], leading to microglial-like cells which do not fully recapitulated bona fide microglia. Nonetheless, in the past few years, several protocols describing the generation of hiPSC-derived microglia properly reproducing their ontogeny have been published [64,162,163,164,165,166,167]. Each protocol has its own methodology, but all of them are based on the production of primitive hematopoietic progenitors similar to yolk sac-derived progenitors during in vivo development, followed by their maturation through the myeloid linage using factors such as macrophage colony-stimulating factor 1 (CSF1), interleukin 34 (IL-34), or transforming growth factor β (TGFβ), key to microglia differentiation and survival [168]. A summary of the methodology to generate hiPSC-derived microglia is depicted in Figure 4A.

All protocols mentioned above generate microglia-like cells that express typical microglial markers, have transcriptomes similar to human primary microglia, and are able to phagocytose. Many of these studies develop microglia that secrete cytokines in response to stimuli [64,163,164,165,166], undergo calcium transients in response to ADP through P2Y purinoreceptor 12 (P2Y12) [163,164], and show engraftment when transplanted into the forebrain of immunodeficient mice [163,167]. As mentioned, microglia survival and maturation are dependent on extracellular signals received from adjacent neurons and glial cells. In attempts to better recreate these signals in vitro, Pandya et al. and Haenseler et al. studies co-cultured microglial precursors with astrocytes and neurons, respectively, with the aim to more faithfully generate cells more similar to human microglia, although these approaches led to more complex and variable culture systems hampering the translation of this technology to serve as platforms for drug discovery.

Although iPSC-derived microglia generated in vitro resemble human microglia, a feature of all these protocols is that generated cells are more similar to fetal that to adult human microglia, representing a hurdle for neurodegenerative disease modeling, where disease-associated mechanisms occur during late adulthood in most cases. Recent investigations have demonstrated that transplantation of hPSC-derived neural cells in AD models is feasible, and it allows for the engraftment and maturation of cells which reproduce the main pathological phenotypes reported in AD patients [169]. Therefore, to increase the level of maturity of hiPSC-derived microglia, recently, a few groups hypothesized that cell maturation should depend on the presence of an in vivo environment and therefore have reported the transplantation of microglial precursors into the developing brain of immunodeficient mice [170,171,172,173].

These studies have confirmed that microglial survival directly depends on CSF1R signaling, with the employment of immunodeficient mice engineered to express the humanized form of the *hCSF1* gene being necessary for these xenograft experiments. These studies reported that microglial precursors colonized and matured within the developing mouse forebrain, acquiring a homeostatic transcriptome signature resembling adult human primary microglia. They also reported that engrafted microglia did not distribute homogenously and presented phenotypic divergences among different brain regions, having a similar heterogeneity as the intrinsic mouse microglia. In addition, engrafted microglia were able to respond to pathological insults such as the presence of Aβ plaques [170], LPS-mediated inflammation [171] or acute demyelination [172]. Notably, Mancuso et al. reported that responses to oligomeric Aβ differed between resident murine microglia and transplanted hPSC-derived microglia [173]. These studies suggest that these xenograft models can be used to better recreate the pathogenic mechanism present in neurodegenerative diseases.

As described above, microglia play a fundamental role in AD pathogenesis which, due to differences between murine and human microglia, can be poorly addressed using mouse models. Therefore, due to the development of specific protocols to generate hiPSC-derived microglia, some studies have already started to evaluate how hiPSC-microglia respond to AD pathological hallmarks. The group of M. Blurton-Jones was the first to describe that hiPSC-microglia derived in vitro were able to phagocytose both Aβ and Tau oligomers both in vitro and in vivo and suffered important gene expression changes after exposure to these pathological insults [163,167]. Using an AD mouse model which allowed the survival of human microglia, they recently reported that in vivo matured hiPSC-microglia expressed genes associated with AD and, when encountered with Aβ plaques, they acquired a distinctive phenotype resembling the disease-associated microglia (DAM) and neurodegeneration-associated microglia (MGnD) phenotypes recently described in AD and other neurodegenerative mouse models [106,107]. These phenotype changes led to the loss of the homeostatic receptor P2RY12 and upregulation of genes such as *APOE*, *TREM2*, or *CD11c*, with specific gene alterations that are unique to the human microglia in contrast to murine microglia, suggesting that this system is best for modeling AD and other neurodegenerative diseases that murine microglia [170].

As the rare variant *R47H* present in the *TREM2* gene increases the risk of having AD by 3–4 fold, few studies have employed hPSC-derived microglia to address the functional consequences of bearing this mutation. Brownjohn et al. first described the derivation of hPSC-derived microglia with Nasu-Hakola disease-associated *TREM2* missense mutations and found that, although lower *TREM2* levels were expressed by mutant cell-derived microglia, this did not affect considerably their phagocytic capacity or response to inflammatory stimuli [174]. On the other hand, Garcia-Reitboeck et al., using similar patient-derived cells but generating microglial precursors using an embryoid body-based methodology, found that these cells presented impaired survival and reduced phagocytic capacity of apoptotic bodies [175]. As relevant to AD, Claes and colleagues evaluated the functional consequences of hPSC-derived microglial-like cells bearing a heterozygous or homozygous deletion of the *TREM2* gene or presenting the *R47H* mutation in one allele. They reported that mutant cells presented a reduced phagocytic capacity of *E. coli* fragments as well as of Aβ plaques using co-cultures of derived microglial-like cells with brain slices from *APP/PSEN1* mice, providing data sustaining that the presence of AD-related *TREM2* mutations made microglia less able to phagocytose Aβ plaques [176]. Notably, this deficiency to phagocytose Aβ plaques has been also reported in vivo [170]. Recently, Piers and colleagues described that, in addition to impaired Aβ phagocytosis, the AD-related *TREM2 R47H* mutation also leads to metabolic impairments in mutant cell-derived microglia, uncovering additional mechanisms involved in *TREM2* mutations [177].

In addition, few studies have addressed the effects of bearing the sporadic AD risk factor *APOE4* allele in hPSC-derived microglia. Microglia derived from *APOE4/4* hiPSC lines showed upregulation of inflammatory genes, an activated phenotype, and a reduced capacity to phagocytose Aβ in comparison with isogenic *APOE3/3* lines [52]. Recently, Konttinen and colleagues reported that hiPSC-derived microglia bearing the *APOE4* allele presented impairment in key microglial functions such as phagocytosis, migration, and metabolic activity but exacerbated cytokine secretion to inflammatory stimuli, suggesting that APOE4 leads to significant functional deficiencies in human microglia [178]. A summary of the findings of the main studies reported using hiPSC-derived microglia is present in Table 3. Moreover, graphical summary of the main pathological findings described in hiPSC-derived AD microglia is represented in Figure 4B.

The relatively recent unveiling of microglial ontogeny has allowed for specific protocols to generate hPSC-derived microglia developed only in the last 3–4 years, with some of them being quite complex, laborious, and inefficient. This, undoubtedly, has impaired the translation of this technology to drug screening efforts. Nonetheless, the last protocols described had reduced complexity and increased the yield to which these cells are generated [167], which should result in the translation of this technology to the drug discovery field. As a proof of concept, drug screening studies using microglia have been performed using primary human or murine microglia. A screening to identify anti-inflammatory compounds in a murine microglial cell line in co-culture with neurons showed that polyphenolic compounds decreased inflammation and protected neurons from microglial damage [179]. Other screening studies in human primary and rat microglia also discovered several anti-inflammatory compounds [180,181]. However, the use of drug screening in hiPSC-derived microglia would have many advantages and will offer the possibility of drug screening in cells derived from AD patients or other neurodegenerative diseases, evaluating the role of these drugs in patient-derived cells. Moreover, this technology will allow co-cultures of hiPSC-derived microglia with hiPSC-derived neurons, astrocytes, or oligodendrocytes, alone or as brain organoids, which will allow not only for assessment of the effects of drugs in microglia but also for confirmation of whether these drugs would have an indirect effect on the survival and pathogenesis of other brain cells

## 6. HiPSC-Derived 3D Cultures and Brain Organoids for AD Modeling and Platform for Screening

### 6.1. Generation of Brain Organoids

**Figure 5:**
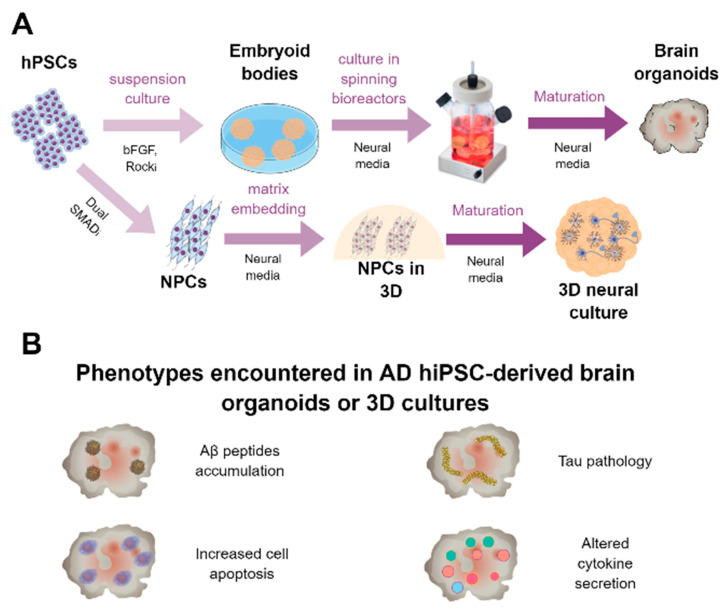
The most used protocol for the generation of brain organoids or 3D neural cell cultures from hPSCs (**A**) and the main phenotypes encountered in organoids or 3D cultures derived from iPSCs of AD patients (**B**). hPSCs: human pluripotent stem cells; iPSCs: induced pluripotent stem cells; bFGF: basic fibroblast growth factor; ROCK: Rho kinase; NPCs: neural precursor cells; Aβ: amyloid beta; AD: Alzheimer’s disease.

In recent years, the generation of 3D cultures formed by neural cells has gained a lot of interest among the research community, as it provides a human platform with higher analogies to the in vivo situation culturing the cells in a monolayer. The generation of brain organoids was first described by Lancaster and colleagues in 2013 [63]. By the generation of embryoid bodies and a default differentiation program towards the neural lineage, they obtained round-shaped 3D structures of up to 4 mm of diameter which expressed specific markers of forebrain regions. In addition, the neurons formed were functional, and the organoids recapitulated the human cortical organization and helped model microcephaly, a condition poorly recapitulated by animal models. Therefore, brain organoids could be used for studying human development and neural diseases [63]. Nonetheless, this system had several limitations such as nutrient and oxygen restrictions in the core of the organoid (leading to notable cell death), early fetal-like phenotype of produced cells, and absence of brain-specific cell populations such as oligodendrocytes or microglia. Part of these drawbacks have been recently addressed by Cakir and colleagues, who transduced a subpopulation of initial hPSCs to overexpress the transcription factor *ETV2*, known to induce human fibroblasts towards endothelium. This led to the formation of brain organoids with vasculature-like structures, which allowed proper supply of nutrients and oxygen to cells within the organoid´s core limiting cell death and, most importantly, this led to a higher degree of cell maturation, analogous to human gestational weeks 16–19 [182]. On the other hand, Ormel et al. developed an organoid protocol following a default neural differentiation program which led to the generation of organoids formed not only by neural cells as neurons, astrocytes, or precursors but also by microglia, which functionally interacted with neurons, elicited immune and phagocyting responses, and resembled mature human tissue-derived microglia, generating a model with very interesting applications in the AD field [183]. Moreover, brain organoids can be utilized to model specific brain structures such as the choroid plexus, allowing the study of drugs able to cross the blood–brain barrier and assessing the clearance of aggregated proteins, key in the study of AD [184]. A summary of the methodology to generate hiPSC-derived 3D neural cultures and brain organoids is depicted in Figure 5A.

### 6.2. Brain Organoids for Modeling AD and to Be Used as a Drug Platform

Choi and colleagues were the first to describe an in vitro 3D culture system able to reproduce AD, although not formed by iPSC-derived cells, [185]. They differentiated human immortalized neural precursor cells into neurons and glia and embedded the cells in gel enriched in brain extracellular matrix proteins (Matrigel), generating a 3D culture. To model AD, they transduced precursor cells with constructs overexpressing the fAD mutations *APP Swe/Lon* and/or *PSEN1 DE9* and analyzed the cultures few weeks later. They found that, on mutant cultures, Aβ accumulated and formed plaques. Notably, cultures composed of mutant cells also presented aggregates of insoluble phosphorylated Tau in the soma and neurites. Treatment of the cells with gamma or beta-secretase specific inhibitors led to a reduction in the levels of Aβ and, remarkably, attenuated Tau pathology. On the other hand, inhibition of the glycogen synthase kinase 3 (GSK3, one of the main kinases involved in Tau phosphorylation) reversed Tau pathology but had no effect on Aβ. This system represented the first in vitro model able to reproduce AD pathology and supported the amyloid hypothesis, which posits that Aβ accumulation is the event that leads to other AD-related pathologies including Tau hyperphosphorylation and accumulation [185]. The group further translated this technology to array platforms which allowed a more homogenous generation of the neurospheroids, where cells presented extensive neurite outgrowth and Aβ and Tau pathologies. Notably, treatment of the spheroids with tested compounds (gamma-secretase inhibitor (compound E), beta-secretase inhibitor (LY2886721), methotrexate, and imatinib) resulted in impaired neurite outgrowth and/or spheroid size, reflecting the fact that this system could be employed for drug testing [186]. Although Aβ aggregation was reproduced in these studies, this was only achieved after *APP* or *PSEN1* mutant isoform overexpression, an experimental condition far from resembling the in vivo situation, highlighting that experimental optimizations are required for more trustworthy disease modeling.

Raja and colleagues found on fAD-derived organoids a higher accumulation of Aβ_40_ and Aβ_42_, an increased expression of phosphorylated Tau, and higher expression of the early endosome antigen 1 (EEA1). Notably, treatment of the organoids with gamma-secretase (compound E) and beta-secretase (beta-secretase inhibitor IV) inhibitors attenuated Aβ and Tau pathology [65]. The Soto lab recently reported the generation of 3D brain organoids from iPSCs of patients with fAD, Down syndrome, or Creutzfeldt–Jakob disease (in addition to murine PSCs), finding that organoids derived from both fAD and Down syndrome patients (but not from Creutzfeldt–Jakob disease patients or murine cells) presented AD-related pathological hallmarks such as accumulation of structures highly reminiscent to amyloid plaques and neurofibrillary tangles which led to higher cell death, reproducing AD features [187].

Some groups generated 3D cultures with different natural or synthetic scaffolds in an attempt to better reproduce brain stiffness in vitro, with the idea that this would better model AD. Zhang and colleagues cultured iPSC-derived NSCs in 3D cultures formed by a self-assembling peptide hydrogel, and in comparison with 2D cultures, they observed that cell cytoskeleton abnormalities linked to AD could be best modeled in 3D, especially when Aβ peptides were added to the culture [188]. Later, Labour and colleagues built a 3D collagen-based scaffold where they grew immortalized neural cell lines in the presence of synthetic Aβ aggregates. They found that, when cells were cultured in scaffolds with pore sizes big enough to allow physical interaction of the cells with Aβ aggregates, it resulted in extensive cell toxicity and neurite dystrophy. Therefore, the authors suggested that this platform could be used to address Aβ-mediated toxicity present in AD [189]. In line with this, recently, Simpson and colleagues demonstrated that the 3D culture in collagen hydrogels accelerated Aβ aggregation and reduced cell toxicity, reinforcing the idea that 3D culture platforms might be suitable for modeling AD-related Aβ accumulation [190]. Recently, the Tanzi group further developed the previously reported 3D culture system [185] and adapted it to be able to model inflammation as well. For this purpose, they cultured human primary and iPSC-derived NSCs embedded in Matrigel in a microfluidic device and overexpressing the *APP K670N/M671L* (Swedish) and *V717I* (London) mutations, reporting Aβ_40_ and Aβ_42_ peptides secretion, increased Tau phosphorylation, and increased expression of inflammatory cytokines and chemokines. In addition, they introduced human-derived microglia on the edges of the structure at different time points and found that microglia migrated to the core of the culture composed by neurons and astrocytes and acquired a reactive and inflammatory phenotype, only in cultures overexpressing fAD *APP* variants. Moreover, this microglial activation led to neuronal toxicity and astrogliosis, phenomena partially governed by IFNg and TLR4-dependent mechanisms. This is, to date, the only study using a 3D in vitro model able to reproduce the main AD pathological hallmarks such as Aβ and Tau pathologies and neuroinflammation [191]. In a further study, they built similar 3D structures but used human-derived neural cells (not iPSC-derived) overexpressing APPSL and/or *PSEN1*ΔE9 proteins from a clonal origin. This allowed them to select clones producing Aβ with different Aβ_42_/Aβ_40_ ratios. Using this system, they demonstrated that Tau pathology was induced not by total Aβ levels but by high Aβ_42_/Aβ_40_ ratios, providing the first proof of this concept in human cells, suggesting that therapies able to diminish this Aβ_42_/Aβ_40_ ratio should be explored for AD [192]. Notably, they suggested that these results might not be reproduced employing iPSC-derived cells as they express lower levels of Aβ_42_/Aβ_40_ and/or do not express mature 4R Tau isoforms.

As a proof that iPSC-derived organoids can be employed for the assessment of candidate drugs, recently, Choi and colleagues generated brain organoids from AD patient and HCs to evaluate the effect of a histone deacetylase 6 (HDAC6) inhibitor, CKD-504, on Tau pathology. The found that exposure of the brain organoid to this compound led to reduced Tau phosphorylation through increase in the degradation pathway of pathological Tau [193].

The hippocampus is involved in the formation of new memories, learning, and emotions and is one of the first regions affected in AD brain [194]. With the purpose of modeling hippocampal pathology present in AD using a human-derived system, Pomeshchik and colleagues, using specific patterning compounds, recently described the generation of hippocampal spheroids from fAD patients with rare mutations in the *APP* (homozygous *p.V717I*) and *PSEN1* (*p.R278K*) genes, which were able to engraft when injected into the hippocampus of *RAG*^−/−^ mice. Familial AD-derived hippocampal spheroids showed increased Aβ42/40 peptide ratios and decreased levels of synaptic proteins. Moreover, they showed other AD-related phenotypes such as Tau hyperphosphorylation, protein aggregation, and protein network alterations but only in *APP* or *PSEN1* mutant spheroids. They also showed a partial recovery of altered pathways mediated by NeuroD1 overexpression, proposing a possible gene therapy-mediated approach to overcome the hippocampal degeneration present in AD [195]. A summary of the findings of the main studies reported using hiPSC-derived 3D neural cultures and brain organoids is present in Table 4. Moreover, graphical summary of the main pathological findings described in hiPSC-derived AD 3D neural cultures and brain organoids is represented in Figure 5B.

All these studies performed using brain organoids/neurospheres suggest that 3D culture may be a better system to reproduce AD in vitro, as it allows for further maturation of the cells and hampers the outflow of secreted proteins, enhancing the aggregation of Aβ and Tau, better reflecting the in vivo situation, and allowing to study the relation between Aβ and Tau pathologies. On the other hand, this tool requires further optimization to reduce variability, even within experimental replicates, and cell composition, as most of the brain organoids generated lack microglia and oligodendrocytes, not representing in this way an optimal system for modeling human brain.

## 7. Concluding Remarks

The complexity of AD pathology, which involves all brain cell types, has precluded the development of specific studies employing hiPSC-derived neural cells. Investigations performed in the past few years have revealed that these AD patient-derived cells are a valid tool to address AD pathogenic pathways and to test the effect of candidate drugs. Nonetheless, initial protocols for the generation of glial cells from hiPSCs were not efficient and robust enough, hampering the obtention of pure population of matured cells in high amounts, a requisite for the employment of these cells in drug discovery programs.

On the other hand, an intrinsic feature of studies employing hiPSCs is the presence of phenotypic variabilities among lines derived from different subjects with the same pathology and even within different clones of the same individual, requiring the employment of several lines per experimental condition to draw robust conclusions. The emergence of CRISPR-Cas9 technology has allowed for an easier implementation of gene-editing tools, allowing simple generation of isogenic lines where the mutation of interest can be erased, introduced, or edited, facilitating the elucidation of phenotypic changes governed by the genotype of interest, which could reduce as well the number of lines to be employed.

Although pharmaceutical and biotechnology companies are very much interested in hPSC technology as it allows for experimentation with human-derived cells, the number of studies using hPSC-derived cells as platforms for drug discovery/validation programs is still low. To facilitate this translation, we think three premises should be compiled: (1) the availability of robust and efficient methods for the generation of the cell(s) of interest, (2) the identification of a robust phenotype to be modified by the candidate compound, and (3) the existence of hPSC-derived robust and homogeneous models of the disease of interest that reproduce most of the phenotypes encountered in patients, overcoming the phenotypic differences that may exist between cells derived from different patients.

The improvement of the lastly developed protocols, together with the emergence of powerful tools for proper in vitro disease modeling as brain organoids, are currently paving the way for a successful implementation of hiPSC technology to precisely reveal AD pathogenesis and to use this system as a better platform for the development of effective drugs able to counteract AD disease progression in the coming years.

## Figures and Tables

**Table 1:** Summary of the findings of the main studies describing the generation of hPSC-derived neurons from patients with Alzheimer’s disease.

Neurons					
Reference	Cell Type Derived	Subjects	Methodology	Main Findings	Drug Evaluation
**Yagi et al. 2011** [33]	Neurons	fAD with PS1 A246E (1) and PS2 N141I (1) mutations	Neurosphere-mediated NPC induction followed by adherent terminal differentiation	Neurons presented higher Aβ_1-42_ production, which was reduced with gamma-secretase inhibitors	Neurons responded to the gamma-secretase inhibitors compounds E and W.
**Israel et al. 2012** [34]	Neurons	HCs (2), fAD with duplication in *APP* (2) and sAD (2)	Embryoid bodies followed by FACS purification	Aberrant secretion of Aβ_1-40_ and increased phospho-Tau and aGSK-3b levels	Neurons responded to beta- but not gamma-secretase inhibitors
**Muratore et al. 2014** [35]	Neurons	fAD with *APP V717I* mutation (2), HCs	Embryoid bodies, followed by neural rosette selection, NPC expansion, and terminal differentiation in monoculture	Altered *APP* processing, secretion of Aβ42 and Aβ38, and increase in total and hyperphosphorylated Tau levels	When neurons were treated with Aβ-specific antibodies, Tau pathology reverted
**Balez et al. 2016** [36]	Neurons	fAD with *PSEN1* P117R mutation (1), sAD with *APOE3/4* genotype (1) and HCs	Neurospheres followed by terminal differentiation in monocultures	AD neurons showed hyperexcitable calcium signaling, elevated levels of nitrite, increased cytotoxicity and apoptosis, reduced neurite length, and increased susceptibility to inflammatory stress	Cells responded to short-term treatment with apigenin, reversing most of these phenotypes
**Nieweg et al. 2015** [37]	Neurons	HCs	Embryoid bodies followed by immunopaning purification	Ab exposure led to a reduction of synapses and electrophysiological activity	-
**Hu et al. 2018** [38]	Neurons	*PS1 Int4* mutation (2), *APP Dup* (2), trisomy 21 (2) and HCs (2)	Dual SMAD inhibition in adherent cultures plus neuronal maturation	Secretome of cells from mutant lines produced inhibition of hippocampal long-term potentiation when injected into rat brains	Antibody blockade of cellular prion protein ameliorated the synaptic loss.
**Chang et al. 2019** [39]	Neurons	fAD with *APP^D678H^* mutation (3), HCs (including an unaffected sibling)	Dual SMAD inhibition in adherent cultures plus neuronal maturation	Accumulation of Aβ_1–42_ and Aβ_1–40_, increased activation of GSK3β, hyperphosphorylation of Tau, and downregulation of synaptophysin	The indole compound NC009-1 partially restored aberrant phenotypes
**Yang et al. 2017** [40]	NPCs and neurons	fAD with *PSEN1* A246E (1) and Ser169del (1) mutations	Generation of neurospheres followed by terminal differentiation in monoculture	Higher levels of Aβ_42_ and Tau phosphorylation and an accelerated neuronal differentiation in mutant cells	-
**Arber et al. 2019** [41]	Neurons and brain organoids	fAD *PSEN1* mutations (5), *APP V717I* (2) and HCs (5)	Neurons: neural induction followed by neuronal maturation in monocultures [25]. Organoids: based as Lancaster et al. 2013 [63]	Mutant cells presented an increased secretion of long Aβ peptides (Ab_40_, Aβ_42_, and Aβ_43_)	-
**Woodruff et al. 2016** [42]	Neurons	Gene-edited iPSC lines with or without PS1 ΔE9, *APP V717F*, and/or *APP* Swedish mutations	NPC generation and expansion, FACS purification, and terminal differentiation	Mutant neurons had defects in the recycling state of endocytosis and soma-to-axon transcytosis of *APP* and lipoproteins	Defects in endocytosis were rescued by beta secretase inhibition
**Martín-Maestro et al. 2017** [43]	Neurons	fAD with *PSEN1* A246E (3) mutations	Dual SMAD inhibition in adherent cultures plus neuronal maturation	Alterations in the autophagic and mitophagy pathways	-
**Martín-Maestro et al. 2019** [44]	NPCs	Gene-edited WT line to incorporate the *PS1 M146L* mutation (1)	Neural induction by dual SMAD inhibition	Mitochondrial respiratory chain defects together with aberrant mitophagy	Treating NPCs with autophagy-stimulating drug bexarotene restored autophagy and compensated mitochondrial abnormalities
**Kwart et al. 2019** [45]	Neurons	Several gene-edited iPSC lines: *APP Swe* (2), *A692G* (2), *V717G* (2), Swe+*M146V* (1), *A673T* (1), *KO* (1), *PSEN1 M146V* (2), *L166P* (2), *M233L* (1), and *A246E* (2)	Dual SMAD inhibition in adherent cultures plus neuronal maturation	Dysregulation of endosomal pathways in mutant cells, which correlated with accumulation of C-terminal fragments produced by the processing of *APP*	This could be rescued by pharmacological modulation of beta-secretase (BACE)
**Birnbaum et al. 2018** [46]	Neurons	sAD (5) and HCs	*NGN2*-based direct generation of neurons from iPSCs	AD cells: increased production of reactive oxygen species (ROS) and higher levels of DNA damage, which did not correlate with Aβ or Tau phosphorylation	-
**Ortíz-Virumbrales et al. 2017** [47]	basal forebrain cholinergic neurons	fAD with *PSEN2 N141I* (2) mutations	Dual SMAD inhibition, FACS selection (CD271), and embryoid body formation followed by neuronal maturation in Brainphys media	Higher production of Aβ42/40 and diminished electrophysiological activity, phenotypes reversed after gene correction	-
**Moreno et al. 2018** [48]	Basal forebrain cholinergic neurons	fAD *PSEN2 N141I* (2), HCs (2), and isogenic gene-corrected controls (2)	Dual SMAD inhibition in adherent cultures plus neuronal maturation in Brainphys media	Mutant cells presented increased Aβ40/Ab42 ratio and altered calcium flux	Addition of insulin to the cultures reduced the increase in Aβ40/Aβ42 ratio and the altered calcium flux
**Kondo et al. 2017** [49]	Neurons	fAD with *PSEN1 G384A* (2), *H163R* (1), *M146L* (1), *A246E* (1), *APP V717L* (1) mutations, sAD (4), fAD gene-corrected lines (2), and HC (4) lines	*NGN2*-based direct generation of neurons from iPSCs	Higher production of Aβ42/40	Tested >1000 compounds on Aβ production and identified six leading compounds with dose-dependent Aβ_42_ reduction, with a combination of three of them (bromocriptine, cromolyn, and topiramate) as the most potent anti-Aβ combinations
**Jin et al. 2018** [50]	Neurons	HC (1)	*NGN2*-based direct generation of neurons from iPSCs	-	AD brain extracts were toxic to the cells. Addition of Aβ-specific blocking antibodies counteracted Aβ-mediated toxicity
**Wang et al. 2018** [51]	Neurons	sAD *APOE4/4* (3), HCs *APOE3/3* (3), isogenic *APOE3/3* (1), and *APOE^−/−^* (1)	Embryoid bodies, neural induction as monocultures, NPC purification and expansion in neurospheres, and terminal maturation in monocultures	*APOE4* neurons had higher levels of Aβ, Tau phosphorylation, and specific degeneration of GABAergic neurons. Converting *APOE4* to *APOE3* by gene editing rescued these phenotypes	Treating the cells with PH002 changed the conformation of *APOE4* to an *APOE3*-like structure reverted the phenotypes, suggesting a possible treatment for sAD.
**Lin et al. 2018** [52]	Neurons, astrocytes, microglia and 3D organoids	sAD (1) and HC (1) lines and isogenic gene-edited controls	Neurons: *NGN2*-based induction. Astrocytes: neural induction followed by sorting for GLAST^+^ cells. Microglia: as described in [64]. Organoids: as described in [65].	*APOE4*+ neurons showed a more mature phenotype with higher presence of synapses and higher secretion of Aβ42. *APOE4* astrocytes and microglia showed a reduced capacity of Aβ uptake.	-
**Meyer et al. 2019** [53]	Neurons and organoids	sAD (5), HCs (5), and gene-edited lines	Neurons: (1) neural induction as embryoid bodies, followed by neuronal maturation and (2) *NGN2*-based direct induction. Organoids: based as Lancaster et al. 2013 [63]	Accelerated neuronal differentiation of *APOE4* iPSC-derived neurons and impaired proliferative capacity of NPCs	-
**Fong et al. 2013** [54]	Neurons	A152T *MAPT* patients (2) and isogenic lines (2)	Neuronal differentiation by dual SMAD inhibition-based methodology	TAU fragmentation and phosphorylation, leading to neurodegeneration. Genetic correction of the mutation restored those phenotypes and homozygous introduction of the mutation exacerbated the phenotypes.	-
**Ehrlich et al. 2015** [66]		*N279K* (1) and V337 M (1) FTDP patients and HCs (3)	Dual SMAD inhibition in embryoid bodies, NPC purification, expansion, and maturation.	Pronounced TAU pathology with increased fragmentation and phosphorylation, decreased neurite extension, and increased oxidative stress. FTD neurons showed an activation of the unfolded protein response and disease-associated gene expression profiles.	
**Wren et al. 2015** [55]	NSCs	FTDP-17 *N279K MAPT* patients (2) and HC (1)	Dual SMAD inhibition in embryoid bodies, NPC purification, and expansion	Increased expression of 4R Tau isoforms, increased cellular stress, and impaired endocytic trafficking, with some of those findings verified using autopsy brains	-
**Iovino et al. 2015** [56]	Neurons	FTDP-17 *MAPT P301L* (2), *N279K* (4), and HCs (3)	Dual SMAD inhibition in adherent cultures plus neuronal maturation	Increase in the 4R/3R ratio of Tau expression, earlier electrophysiological maturation, and an increase in Tau phosphorylation	-
**Imamura et al. 2016** [57]	Neurons	Patients with I10+14C (1) and R406W (1) mutations and gene-corrected isogenic lines	*NGN2*-based direct generation of neurons from iPSCs	Diseased neurons showed dysregulation of the augmentation of Ca^2+^ transients evoked by electrical stimulation which led to the release of misfolded Tau and cell death	Chemogenetic or pharmacological control of Ca^2+^ influx by the introduction of designer receptors exclusively activated by designer drugs or by treatment with glutamate receptor blockers attenuated misfolded tau accumulation and neuronal death
**Nakamura et al. 2019** [58]	Neurons	Patients with R406W mutations (2) and gene-corrected isogenic lines	Generation of neural organoids followed by singularization and neuronal culture	Notable Tau fragmentation and mislocation, which led axons to display morphological and functional abnormalities that could be rescued by microtubule stabilization	Morphological and functional abnormalities that could be rescued by a microtubule stabilizer (epothilone D)
**García-León et al.** [59]	Neurons	hiPSCs with the introduction of the *N279K*, *P301L*, and E10+16 mutations.	Dual SMAD inhibition in adherent cultures plus neuronal maturation	Mutant neurons expressed higher levels of 4R Tau, higher phosphorylation and aggregation of Tau, an increased electrophysiological activity, deficiencies in neurite outgrowth, aberrant sequence of differentiation to cortical neurons, activation of stress pathways, shift toward GABAergic identity, and an upregulation of neurodegenerative pathways.	-
**Medda et al. 2016** [60]	Neurons	HCs (2)	NPC generation by dual SMAD inhibition followed by neuronal maturation in 3D	Tau aggregation when pathogenic *P301L* Tau is overexpressed in the presence of K18 fibrils	Platform compatible with assessing compounds able to reduce Tau aggregation
**Wang et al. 2017** [61]	Neurons	HCs (2)	*NGN2*-based direct generation of neurons from iPSCs	Developed a platform to test Ta lowering in a high-throughput manner	Among the 1280 tested compounds, three reduced Tau content without cell toxicity
**Van der Kant et al. 2019** [62]	NPCs, neurons and astrocytes	fAD, sAD, gene-edited lines, and HCs	NPCs: dual SMAD inhibition followed by FACS sorting. Neurons: neuronal maturation and FACS purification. Astrocytes: sphere-mediated astrocytic induction	Increased Tau phosphorylation and Aβ secretion	Tested >1600 compounds, finding several hits involved in cholesterol pathway. Identified cholesteryl esters (CE), the storage product of excess cholesterol, as upstream regulators of pTau and Aβ secretion, suggesting a therapeutic possibility for AD.

AD: Alzheimer’s disease; PS1: presenilin 1; PS2: presenilin 2; *APP*: amyloid precursor protein; Aβ: amyloid beta; HC: healthy control; sAD: sporadic Alzheimer’s disease; fAD: familial Alzheimer’s disease.

**Table 2:** Summary of the findings of the main studies describing the generation of hPSC-derived astrocytes from patients with Alzheimer’s disease.

Astrocytes					
Reference	Cell Type Derived	Subjects	Methodology	Main Findings	Drug Evaluation
**Kondo et al. 2013** [123].	iPSC-derived astrocytes and neurons	HC (1 subject), sAD (1 subject), and fAD (*APP E693Δ*, 1 subject).	Embryoid bodies, NPC expansion, astrocyte purification by attachment, and terminal differentiation with FBS.	Intracellular Aβ oligomer accumulation. Upregulation of ER and UPR stress.	BSI: reduction of Aβ oligomers and stress. DHA: alleviation of stress. DBM14-26 and NSC23766: no improvement of stress
**Oksanen et al. 2017** [124]	iPSC-derived astrocytes	HC (3), fAD *PSEN1 DE9* (2), pre-symptomatic *PSEN1 DE9* (1), and *PSEN1 DE9* gene-corrected (2)	Embryoid bodies, NPC expansion, glial induction, and astrocyte maturation with BMP4 and CNTF.	*PSEN1*-mutants: Increased Aβ_1-42_ secretion. Reduced capacity of Aβ internalization. Lack of proper support of neurons and induction of toxicity. Altered cytokine secretion.	-
**Jones et al. 2017** [125]	iPSC-derived astrocytes	HCs, fAD *PSEN1 M146L* (1), and sAD *APOE4*^+/+^ (1)	Dual SMAD inhibition; NPC expansion; glial induction; and astrocyte maturation with FGF2, CNTF, BMP2, EGF, and insulin.	Astrocyte-specific deficiencies in both fAD and sAD cases: Simplified morphology and smaller cell volume and area. Accumulation of astrocytic markers in the nuclei. Altered basal inflammatory cytokine production	-
**Zhao et al. 2017** ([126]	iPSC-derived astrocytes and neurons	Cognitively normal *APOE3*^+/+^ and *APOE4*^+/+^ individuals	Embryoid bodies; NPC expansion; glial induction; and astrocyte maturation with BMP4, CNTF, heregulin-b, and FBS.	In *APOE4^+/+^*-derived astrocytes, APOE with a less lipidation status. Defective neuronal support	-
**Hallmann et al. 2017** [127]	iPSC-derived astrocytes	HCs, FTD *TAU N279K* (2), and *N279K* gene-corrected (2)	*SOX10*-induction of astrocytes	Aberrant expression of 4R TAU. Larger cell volume and reactivity. Upregulation in ROS and UPR stress. Toxic to neurons	-

AD: Alzheimer’s disease; PS1: presenilin 1; PS2: presenilin 2; *APP*: amyloid precursor protein; Aβ: amyloid beta; HC: healthy control; sAD: sporadic Alzheimer’s disease; fAD: familial Alzheimer’s disease.

**Table 3:** Summary of the findings of the main studies describing the generation of hPSC-derived microglia from patients with Alzheimer’s disease.

Microglia					
Reference	Cell Type Derived	Subjects	Methodology	Main Findings	Drug Evaluation
**Muffat et al. 2016** [64]	Microglia	HCs (8), adrenoleukodystrophy (4), adrenomyeloneuropathy (3), Rett syndrome (1), or AD (3)	Embryoid body-based using a defined neural medium supplemented with CSF1 and IL-34 to generate myeloid progenitors and later microglia	Resembles primary microglia at the transcriptome and functional (phagocytosis, cytokine secretion, and response to injury) levels and interacted with neurons and glial cells	-
**Pandya et al. 2017** [162]	Microglia	HCs (2)	Induction towards myeloid progenitors, FACS purification, and maturation in the presence of hPSC-derived astrocytes	Murine and human microglia resembled primary microglia at the transcriptome level as well as functionally (phagocytosis, ROS production, and improved outcomes of mice bearing brain gliomas).	-
**Abud et al. 2017** [163]	Microglia	HCs (10)	Induction towards hematopoietic progenitors, CD43+ enrichment, and maturation in the presence of MCSF, IL34, TGFb1, CD200, and CX3CL1	Generated microglial cells resembling human fetal and adult microglia at the transcriptome level and responded to inflammatory stimuli, phagocytose Aβ, and phosphorylated Tau	-
**Douvaras et al. 2017** [164]	Microglia	HCs (11), Parkinson’s (3), multiple sclerosis (1), and AD (1)	Induction towards hematopoietic progenitors, CD14+/CX3CR1+ enrichment, and maturation in the presence of GM-CSF and IL34.	Similar to primary microglia at the transcriptome level and cytokine expression profile, able to phagocytose and responded to ADP.	-
**Haenseler et al. 2017** [165]	Microglia	HCs (6)	Induction towards hematopoietic progenitors in embryoid bodies, collection of hematopoietic progenitors, and maturation in the presence of GM-CSF and IL34 and in co-culture with neurons	Similar to primary microglia at the transcriptome level and cytokine expression profile, able to phagocytose, activated with LPS, and surveyed the environment as the primary microglia.	-
**Takata et al. 2017** [166]	Murine and human microglia and other macrophages	Parkinson’s (1) and familial Mediterranean fever (2)	Differentiation towards early macrophages following molecular cues present during yolk sac hematopoiesis and microglial maturation in co-culture with neurons	Generated cells resembled fetal microglia, interacted with neurons, responded to injury, were able to phagocytose, and synthesized cytokines	-
**McQuade et al. 2018** [167]	Microglia	HCs (5)	Induction towards hematopoietic CD43+ progenitors and maturation in the presence of MCSF, IL34, TGFb1, CD200, and CX3CL1	This paper describes a simplified and more efficient protocol compared to the previously developed by the group [163], which results in microglia with the same features as the previous report.	-
**Hasselmann et al. 2019** [170]	Microglia precursors	HC (1) and gene-edited *TREM2 R47H* (1)	Differentiation towards microglial progenitors as previously described [167] and transplanted in immunodeficient mice	Transplanted cells better resembled primary human microglia that cultured cells, perform homeostatic functions, responded to injury, and reacted to Aβ plaques with a differential transcriptome signature compared to murine microglia	-
**Svoboda et al. 2019** [171]	Microglial precursors and mature microglia	HC (1)	Differentiation towards microglial progenitors and matured cells according to Douvaras et al. 2017 [164] and transplanted in immunodeficient mice	Transplanted cells colonized the whole brain of hCSF1-expressing mice. Injected precursors acquired a mature microglial phenotype after in vivo maturation resembling human primary microglia in homeostatic state.	-
**Xu et al. 2020** [172]	Primitive macrophage progenitors	HCs (2)	Differentiation towards macrophage progenitors as previously described [165] and transplanted into immunodeficient mice	Transplanted cells colonized the whole brain of hCSF1-expressing mice. Injected precursors acquired a mature microglial homeostatic phenotype as adult human microglia, expressed neurodegenerative disease-associated genes, and responded to acute demyelination.	-
**Mancuso et al. 2019** [173]	Monocyte-derived microglia from hPSCs	HC (1)	First, monocytes were differentiated from hPSCs as described [176], and then, monocytes were induced towards neural lineage in neural medium.	Embryonic stem cell-derived microglia survive and integrate in mouse brain and mimic primary human cells at the transcriptome level. Human ESC-derived and host mouse microglia display a divergent response to oligomeric amyloid-β.	-
**Brownjohn et al. 2018** [174]	Microglia	HCs (3), Nasu-Hakola disease patients (2), or unaffected family members (2)	Derivation of primitive macrophage precursors, and microglia and microglia maturation in the presence of GM-CSF and IL34.	Lower *TREM2* levels were expressed by mutant cell-derived microglia, but this did not affect considerably their phagocytic capacity or response to inflammatory stimuli	-
**Garcia-Reitboeck et al. 2018** [175]	Microglia	HCs (4), Nasu-Hakola disease patients (2), or unaffected family members (2)	Derivation of primitive macrophage precursors through an embryoid body-based method and microglia maturation.	*TREM2* mutant cells presented impaired survival and reduced phagocytic capacity of apoptotic bodies.	-
**Claes et al. 2018** [176]	Monocyte-derived microglia from hPSCs	HC (1) and gene-edited lines (3)	First, monocytes were differentiated from hPSCs and, then, monocytes were induced towards neural lineage in neural medium.	Mutant cells presented a reduced phagocytic capacity of *E. coli* fragments as well as of Aβ plaques using co-cultures of derived microglial-like cells with brain slices from *APP*/PS1 mice.	-
**Piers et al. 2020** [177]	Microglia	HCs and diseased or gene-edited lines with R47H, T66M, or W50C mutations	As previously described [158]	The AD-related *TREM2* R47H mutation produced impaired Aβ phagocytosis and led to metabolic impairments	-
**Lin et al. 2018** [52]	Neurons, astrocytes, microglia and organoids	HC (1), sAD (1), and gene-edited lines with *APOE3/3* and *APOE4/4* genotypes	Microglia was generated as in Muffat et al. [64].	Microglia derived from *APOE4*/4 hiPSC lines showed upregulation of inflammatory genes, an activated phenotype, and a reduced capacity to phagocytose Aβ in comparison with isogenic *APOE3/3* lines.	-
**Konttinen et al. 2019** [178]	Microglia	HCs (5), *APOE4* sAD (3), *PSEN1ΔE9* (4), and *APP Swedish* (2).	Small molecule generation of myeloid progenitors and maturation in the presence of MSCF and IL-34.	*APOE4* microglia had impairment in key microglial functions such as phagocytosis, migration, and metabolic activity but exacerbated cytokine secretion to inflammatory stimuli. *APP* or *PSEN1* mutations had little impact on microglial functionality.	-

AD: Alzheimer’s disease; PS1: presenilin 1; PS2: presenilin 2; *APP*: amyloid precursor protein; Aβ: amyloid beta; HC: healthy control; sAD: sporadic Alzheimer’s disease; fAD: familial Alzheimer’s disease.

**Table 4:** Summary of the findings of the main studies describing the generation of hPSC-derived brain organoids and neural 3D cultures from patients with Alzheimer´s disease.

Organoids					
Reference	Cell Type Derived	Subjects	Methodology	Main Findings	Drug Evaluation
**Choi et al. 2014** [185]	An immortalized human neural stem cell line (ReNcell)	Lentiviral-mediated overexpression of the fAD mutations *APP Swe/Lon* and/or *PSEN1 DE9*	Neural differentiation in a defined medium with cells embedded in a gel matrix (Matrigel)	Able to model AD in vitro, as this system led to Aβ accumulation, which subsequently induced Tau pathology	Beta-secretase inhibitors (DAPT, compound E and b-secretase inhibitor IV) or gamma-secretase inhibition (SGSM41) reverted Aβ and Tau pathology
**Jorfi et al. 2018** [186]	An immortalized human neural stem cell line (ReNcell)	An immortalized human neural stem cell line (ReNcell)	Neural differentiation in a defined medium with cells embedded in a gel matrix (Matrigel) and these neurospheroids allocated within defined arrays	Cells presented extensive neurite outgrowth, and Aβ and Tau pathologies	Treatment of the spheroids with five tested compounds (gamma–secretase inhibitor (compound E), beta–secretase inhibitor (LY2886721), methotrexate, and imatinib) resulted in impaired neurite outgrowth and/or spheroid size.
**Raja et al. 2016** [65]	iPSC-derived neural cells	HCs (2), fAD *APPdup* (2), and fAD *PSEN1 M146I* (1) *PSEN1 A264E* (1).	Embryoid bodies formation, neural induction, and neural maturation in neural medium with Matrigel	higher accumulation of Aβ40 and Aβ 42, an increased expression of phosphorylated Tau, and higher expression of the early endosome antigen 1 (EEA1).	Treatment of the organoids with gamma-secretase (compound E) and beta-secretase (beta-secretase inhibitor IV) inhibitors attenuated Aβ and Tau pathology
**Gonzalez et al. 2018** [187]	iPSC-derived neural cells	HC (1), fAD *PSEN1* A246E (1), Down syndrome (1), Creutzfeldt–Jakob disease (2), mouse ESCs, and iPSCs.	Brain organoid generation according to Lancaster et al. 2013 [63].	Organoids derived from both fAD and Down syndrome patients presented accumulation of amyloid-like plaques and neurofibrillary tangles, which led to higher cell death	-
**Zhang et al. 2014** [188]	iPSC-derived neural cells	HCs (1)	Neural cells were cultured in self-assembling peptide hydrogels or 2D and allowed to mature by growth factor withdrawal.	Cytoskeleton abnormalities linked to AD could be best modeled in 3D, especially when Aβ peptides were added to the culture	-
**Labour et al. 2016** [189]	Immortalized human cell lines (PC12 and SH-SY6Y)	Non-demented subjects	Neural cell cultured in 3D collagen-based scaffolds	Physical contacts between cells and Aβ aggregates resulted in cell toxicity and neurite dystrophy	-
**Park et al. 2018** [191]	Immortalized human neural stem cell line (ReNcell), human immortalized microglia cell line, and iPSC-derived neural cells	Immortalized human neural stem cell line (ReNcell), human immortalized microglia cell line, and iPSC-derived neural cells	Neural differentiation in defined medium with cells embedded in a gel matrix (Matrigel) with these neurospheroids allocated into a microfluidic device	Aβ_40_ and Aβ_42_ secretion, Tau phosphorylation, and expression of inflammatory cytokines and chemokines. Introduction of microglia led to neurotoxicity and astrogliosis	-
**Kwak et al. 2020** [192]	Immortalized human neural stem cell line (ReNcell)	Immortalized human neural stem cell line (ReNcell) overexpressing APPSL and/or PS1ΔE9 isoforms	Neural differentiation in a defined medium with cells embedded in a gel matrix (Matrigel)	Tau pathology was demonstrated to be induced in cells with a high Aβ_42_/Aβ_40_ ratio	Treatment with BPN-15606, a γ-secretase modulator, which reduced Aβ42/40, effectively reducing p-Tau accumulation.
**Choi et al. 2020** [193]	iPSC-derived neural cells	HC (1) and AD (1)	Embryoid body formation, neural induction, and neural maturation in a neural medium	Increased Tau phosphorylation	A HDAC6 inhibitor, CKD-504, led to reduced Tau phosphorylation by increasing the degradation of pathological Tau
**Pomeshchik et al. 2020** [195]	Hippocampal spheroids formed by iPSC-derived neural cells	HCs, fAD with *APP p.V717I* (1) and *PS1 p.R278K* genes (1)	Embryoid body formation, neural induction, and neural maturation in neural medium with specific morphogens	increased Aβ42/40 peptide ratios and decreased levels of synaptic proteins	NeuroD1 overexpression partially restored aberrant phenotypes

AD: Alzheimer’s disease; PS1: presenilin 1; PS2: presenilin 2; *APP*: amyloid precursor protein; Aβ: amyloid beta; HC: healthy control; sAD: sporadic Alzheimer’s disease; fAD: familial Alzheimer’s disease.

## References

[1] Winblad B., Amouyel P., Andrieu S., Ballard C., Brayne C., Brodaty H., Cedazo-Minguez A., Dubois B., Edvardsson D., Feldman H. (2016). Defeating Alzheimer’s disease and other dementias: A priority for European science and society. Lancet Neurol..

[2] Serrano-Pozo A., Frosch M.P., Masliah E., Hyman B.T. (2011). Neuropathological alterations in Alzheimer disease. Cold Spring Harb. Perspect. Med..

[3] Deture M.A., Dickson D.W. (2019). The neuropathological diagnosis of Alzheimer’s disease. Mol. Neurodegener..

[4] Sims R., Hill M., Williams J. (2020). The multiplex model of the genetics of Alzheimer’s disease. Nat. Neurosci..

[5] Guerreiro R., Wojtas A., Bras J., Carrasquillo M., Rogaeva E., Majounie E., Cruchaga C., Sassi C., Kauwe J.S.K., Younkin S. (2013). TREM2 variants in Alzheimer’s disease. N. Engl. J. Med..

[6] Bettens K., Sleegers K., Van Broeckhoven C. (2013). Genetic insights in Alzheimer’s disease. Lancet Neurol..

[7] Selkoe D.J., Hardy J. (2016). The amyloid hypothesis of Alzheimer’s disease at 25 years. EMBO Mol. Med..

[8] Mehta D., Jackson R., Paul G., Shi J., Sabbagh M. (2017). Why do trials for Alzheimer’s disease drugs keep failing? A discontinued drug perspective for 2010–2015. Expert Opin. Investig. Drugs.

[9] Arranz A.M., De Strooper B. (2019). The role of astroglia in Alzheimer’s disease: Pathophysiology and clinical implications. Lancet Neurol..

[10] Heneka M.T., Carson M.J., El Khoury J., Landreth G.E., Brosseron F., Feinstein D.L., Jacobs A.H., Wyss-Coray T., Vitorica J., Ransohoff R.M. (2015). Neuroinflammation in Alzheimer’s disease. Lancet Neurol..

[11] McQuade A., Blurton-Jones M. (2019). Microglia in Alzheimer’s Disease: Exploring How Genetics and Phenotype Influence Risk. J. Mol. Biol..

[12] Sarlus H., Heneka M.T. (2017). Microglia in Alzheimer’s disease. J. Clin. Investig..

[13] Hansen D.V., Hanson J.E., Sheng M. (2018). Microglia in Alzheimer’s disease. J. Cell Biol..

[14] Shi Y., Holtzman D.M. (2018). Interplay between innate immunity and Alzheimer disease: APOE and TREM2 in the spotlight. Nat. Rev. Immunol..

[15] Ulland T.K., Colonna M. (2018). TREM2—A key player in microglial biology and Alzheimer disease. Nat. Rev. Neurol..

[16] Webers A., Heneka M.T., Gleeson P.A. (2020). The role of innate immune responses and neuroinflammation in amyloid accumulation and progression of Alzheimer’s disease. Immunol. Cell Biol..

[17] Navarro V., Sanchez-Mejias E., Jimenez S., Muñoz-Castro C., Sanchez-Varo R., Davila J.C., Vizuete M., Gutierrez A., Vitorica J. (2018). Microglia in Alzheimer’s Disease: Activated, Dysfunctional or Degenerative. Front. Aging Neurosci..

[18] King A. (2018). The search for better animal models of Alzheimer’s disease. Nature.

[19] Garcia-Leon J.A., Vitorica J., Gutierrez A. (2019). Use of human pluripotent stem cell-derived cells for neurodegenerative disease modeling and drug screening platform. Future Med. Chem..

[20] Calderon-Garcidueñas A.L., Duyckaerts C. (2018). Alzheimer disease. Handb. Clin. Neurol..

[21] Braak H., Alafuzoff I., Arzberger T., Kretzschmar H., Tredici K. (2006). Staging of Alzheimer disease-associated neurofibrillary pathology using paraffin sections and immunocytochemistry. Acta Neuropathol..

[22] Sanchez-Mejias E., Nuñez-Diaz C., Sanchez-Varo R., Gomez-Arboledas A., Garcia-Leon J.A., Fernandez-Valenzuela J.J., Mejias-Ortega M., Trujillo-Estrada L., Baglietto-Vargas D., Moreno-Gonzalez I. (2020). Distinct disease-sensitive GABAergic neurons in the perirhinal cortex of Alzheimer’s mice and patients. Brain Pathol..

[23] Gaspard N., Bouschet T., Hourez R., Dimidschstein J., Naeije G., Van Den J., Passante L., Schiffmann S.N., Gaillard A., Espuny-camacho I. (2008). An intrinsic mechanism of corticogenesis from embryonic stem cells. Nature.

[24] Chambers S.M., Fasano C.A., Papapetrou E.P., Tomishima M., Sadelain M., Studer L. (2009). Highly efficient neural conversion of human ES and iPS cells by dual inhibition of SMAD signaling. Nat. Biotechnol..

[25] Shi Y., Kirwan P., Smith J., Robinson H.P.C., Livesey F.J. (2012). Human cerebral cortex development from pluripotent stem cells to functional excitatory synapses. Nat. Neurosci..

[26] Kikuchi T., Morizane A., Doi D., Magotani H., Onoe H., Hayashi T., Mizuma H., Takara S., Takahashi R., Inoue H. (2017). Human iPS cell-derived dopaminergic neurons function in a primate Parkinson’s disease model. Nature.

[27] Tao Y., Zhang S.C. (2016). Neural Subtype Specification from Human Pluripotent Stem Cells. Cell Stem Cell.

[28] Ambasudhan R., Talantova M., Coleman R., Yuan X., Zhu S., Lipton S.A., Ding S. (2011). Direct reprogramming of adult human fibroblasts to functional neurons under defined conditions. Cell Stem Cell.

[29] Pang Z.P., Yang N., Vierbuchen T., Ostermeier A., Fuentes D.R., Yang T.Q., Citri A., Sebastiano V., Marro S., Südhof T.C. (2011). Induction of human neuronal cells by defined transcription factors. Nature.

[30] Caiazzo M., Dell’Anno M.T., Dvoretskova E., Lazarevic D., Taverna S., Leo D., Sotnikova T.D., Menegon A., Roncaglia P., Colciago G. (2011). Direct generation of functional dopaminergic neurons from mouse and human fibroblasts. Nature.

[31] Zhang Y., Pak C.H., Han Y., Ahlenius H., Zhang Z., Chanda S., Marro S., Patzke C., Acuna C., Covy J. (2013). Rapid single-step induction of functional neurons from human pluripotent stem cells. Neuron.

[32] Chanda S., Ang C.E., Lee Q.Y., Ghebrial M., Haag D., Shibuya Y., Wernig M., Südhof T.C. (2019). Direct Reprogramming of Human Neurons Identifies MARCKSL1 as a Pathogenic Mediator of Valproic Acid-Induced Teratogenicity. Cell Stem Cell.

[33] Yagi T., Ito D., Okada Y., Akamatsu W., Nihei Y., Yoshizaki T., Yamanaka S., Okano H., Suzuki N. (2011). Modeling familial Alzheimer’s disease with induced pluripotent stem cells. Hum. Mol. Genet..

[34] Israel M.A., Yuan S.H., Bardy C., Reyna S.M., Mu Y., Herrera C., Hefferan M.P., Van Gorp S., Nazor K.L., Boscolo F.S. (2012). Probing sporadic and familial Alzheimer’s disease using induced pluripotent stem cells. Nature.

[35] Muratore C.R., Rice H.C., Srikanth P., Callahan D.G., Shin T., Benjamin L.N.P., Walsh D.M., Selkoe D.J., Young-Pearse T.L. (2014). The familial alzheimer’s disease APPV717I mutation alters APP processing and Tau expression in iPSC-derived neurons. Hum. Mol. Genet..

[36] Balez R., Steiner N., Engel M., Muñoz S.S., Lum J.S., Wu Y., Wang D., Vallotton P., Sachdev P., O’Connor M. (2016). Neuroprotective effects of apigenin against inflammation, neuronal excitability and apoptosis in an induced pluripotent stem cell model of Alzheimer’s disease. Sci. Rep..

[37] Nieweg K., Andreyeva A., Van Stegen B., Tanriöver G., Gottmann K. (2015). Alzheimer’s disease-related amyloid-β induces synaptotoxicity in human iPS cell-derived neurons. Cell Death Dis..

[38] Hu N., Corbett G.T., Moore S., Walsh D.M., Livesey F.J., Rowan M.J., Hu N., Corbett G.T., Moore S., Klyubin I. (2018). Extracellular Forms of A b and Tau from iPSC Models of Alzheimer ’s Disease Disrupt Synaptic Plasticity Report Extracellular Forms of A b and Tau from iPSC Models of Alzheimer’s Disease Disrupt Synaptic Plasticity. Cell Rep..

[39] Chang K.H., Lee-Chen G.J., Huang C.C., Lin J.L., Chen Y.J., Wei P.C., Lo Y.S., Yao C.F., Kuo M.W., Chen C.M. (2019). Modeling Alzheimer’s Disease by Induced Pluripotent Stem Cells Carrying APP D678H Mutation. Mol. Neurobiol..

[40] Yang J., Zhao H., Ma Y., Shi G., Song J., Tang Y., Li S., Li T., Liu N., Tang F. (2017). Early pathogenic event of Alzheimer’s disease documented in iPSCs from patients with PSEN1 mutations. Oncotarget.

[41] Arber C., Toombs J., Lovejoy C.C., Ryan N.S., Paterson R.W., Willumsen N., Gkanatsiou E., Portelius E., Blennow K., Heslegrave A. (2019). Familial Alzheimer’s disease patient-derived neurons reveal distinct mutation-specific effects on amyloid beta. Mol. Psychiatry.

[42] Woodruff G., Reyna S.M., Dunlap M., Van Der Kant R., Callender J.A., Young J.E., Roberts E.A., Goldstein L.S.B. (2016). Defective Transcytosis of APP and Lipoproteins in Human iPSC-Derived Neurons with Familial Alzheimer’s Disease Mutations. Cell Rep..

[43] Martín-Maestro P., Gargini R., Sproul A.A., García E., Antón L.C., Noggle S., Arancio O., Avila J., García-Escudero V. (2017). Mitophagy failure in fibroblasts and iPSC-derived neurons of alzheimer’s disease-associated presenilin 1 mutation. Front. Mol. Neurosci..

[44] Martín-Maestro P., Sproul A., Martinez H., Paquet D., Gerges M., Noggle S., Starkov A.A. (2019). Autophagy Induction by Bexarotene Promotes Mitophagy in Presenilin 1 Familial Alzheimer’s Disease iPSC-Derived Neural Stem Cells. Mol. Neurobiol..

[45] Kwart D., Gregg A., Scheckel C., Murphy E., Paquet D., Duffield M., Fak J., Olsen O., Darnell R., Tessier-Lavigne M. (2019). A Large Panel of Isogenic APP and PSEN1 Mutant Human iPSC Neurons Reveals Shared Endosomal Abnormalities Mediated by APP β-CTFs, Not Aβ. Neuron.

[46] Birnbaum J.H., Wanner D., Gietl A.F., Saake A., Kündig T.M., Hock C., Nitsch R.M., Tackenberg C. (2018). Oxidative stress and altered mitochondrial protein expression in the absence of amyloid-β and tau pathology in iPSC-derived neurons from sporadic Alzheimer’s disease patients. Stem Cell Res..

[47] Ortiz-Virumbrales M., Moreno C.L., Kruglikov I., Marazuela P., Sproul A., Jacob S., Zimmer M., Paull D., Zhang B., Schadt E.E. (2017). CRISPR/Cas9-Correctable mutation-related molecular and physiological phenotypes in iPSC-derived Alzheimer’s PSEN2 N141I neurons. Acta Neuropathol. Commun..

[48] Moreno C.L., Guardia L.D., Shnyder V., Ortiz-Virumbrales M., Kruglikov I., Zhang B., Schadt E.E., Tanzi R.E., Noggle S., Buettner C. (2018). IPSC-derived familial Alzheimer’s PSEN2 N141I cholinergic neurons exhibit mutation-dependent molecular pathology corrected by insulin signaling. Mol. Neurodegener..

[49] Kondo T., Imamura K., Funayama M., Tsukita K., Miyake M., Ohta A., Woltjen K., Nakagawa M., Asada T., Arai T. (2017). iPSC-Based Compound Screening and In Vitro Trials Identify a Synergistic Anti-amyloid β Combination for Alzheimer’s Disease. Cell Rep..

[50] Jin M., O’Nuallain B., Hong W., Boyd J., Lagomarsino V.N., O’Malley T.T., Liu W., Vanderburg C.R., Frosch M.P., Young-Pearse T. (2018). An in vitro paradigm to assess potential anti-Aβ antibodies for Alzheimer’s disease. Nat. Commun..

[51] Wang C., Najm R., Xu Q., Jeong D.E., Walker D., Balestra M.E., Yoon S.Y., Yuan H., Li G., Miller Z.A. (2018). Gain of toxic apolipoprotein E4 effects in human iPSC-derived neurons is ameliorated by a small-molecule structure corrector article. Nat. Med..

[52] Lin Y.-T.T., Seo J., Gao F., Feldman H.M., Wen H.-L.L., Penney J., Cam H.P., Gjoneska E., Raja W.K., Cheng J. (2018). APOE4 Causes Widespread Molecular and Cellular Alterations Associated with Alzheimer’s Disease Phenotypes in Human iPSC-Derived Brain Cell Types. Neuron.

[53] Meyer K., Feldman H.M., Lu T., Drake D., Lim E.T., Ling K.-H., Bishop N.A., Pan Y., Seo J., Lin Y.-T. (2019). REST and Neural Gene Network Dysregulation in iPSC Models of Alzheimer’s Disease. Cell Rep..

[54] Fong H., Wang C., Knoferle J., Walker D., Balestra M.E., Tong L.M., Leung L., Ring K.L., Seeley W.W., Karydas A. (2013). Genetic correction of tauopathy phenotypes in neurons derived from human induced pluripotent stem cells. Stem Cell Rep..

[55] Wren M.C., Zhao J., Liu C.-C., Murray M.E., Atagi Y., Davis M.D., Fu Y., Okano H.J., Ogaki K., Strongosky A.J. (2015). Frontotemporal dementia-associated N279K tau mutant disrupts subcellular vesicle trafficking and induces cellular stress in iPSC-derived neural stem cells. Mol. Neurodegener..

[56] Iovino M., Agathou S., González-Rueda A., Del Castillo Velasco-Herrera M., Borroni B., Alberici A., Lynch T., O’Dowd S., Geti I., Gaffney D. (2015). Early maturation and distinct tau pathology in induced pluripotent stem cell-derived neurons from patients with *MAPT* mutations. Brain.

[57] Imamura K., Sahara N., Kanaan N.M., Tsukita K., Kondo T., Kutoku Y., Ohsawa Y., Sunada Y., Kawakami K., Hotta A. (2016). Calcium dysregulation contributes to neurodegeneration in FTLD patient iPSC-derived neurons. Sci. Rep..

[58] Nakamura M., Shiozawa S., Tsuboi D., Amano M., Watanabe H., Maeda S., Kimura T., Yoshimatsu S., Kisa F., Karch C.M. (2019). Pathological Progression Induced by the Frontotemporal Dementia-Associated R406W Tau Mutation in Patient-Derived iPSCs. Stem Cell Rep..

[59] García-León J.A., Cabrera-Socorro A., Eggermont K., Swijsen A., Terryn J., Fazal R., Nami F., Ordovás L., Quiles A., Lluis F. (2018). Generation of a human induced pluripotent stem cell–based model for tauopathies combining three microtubule-associated protein TAU mutations which displays several phenotypes linked to neurodegeneration. Alzheimer’s Dement..

[60] Medda X., Mertens L., Versweyveld S., Diels A., Barnham L., Bretteville A., Buist A., Verheyen A., Royaux I., Ebneth A. (2016). Development of a Scalable, High-Throughput-Compatible Assay to Detect Tau Aggregates Using iPSC-Derived Cortical Neurons Maintained in a Three-Dimensional Culture Format. J. Biomol. Screen..

[61] Wang C., Ward M.E., Chen R., Liu K., Tracy T.E., Chen X., Xie M., Sohn P.D., Ludwig C., Meyer-Franke A. (2017). Scalable Production of iPSC-Derived Human Neurons to Identify Tau-Lowering Compounds by High-Content Screening. Stem Cell Rep..

[62] van der Kant R., Langness V.F., Herrera C.M., Williams D.A., Fong L.K., Leestemaker Y., Steenvoorden E., Rynearson K.D., Brouwers J.F., Helms J.B. (2019). Cholesterol Metabolism Is a Druggable Axis that Independently Regulates Tau and Amyloid-β in iPSC-Derived Alzheimer’s Disease Neurons. Cell Stem Cell.

[63] Lancaster M.A., Renner M., Martin C.-A., Wenzel D., Bicknell L.S., Hurles M.E., Homfray T., Penninger J.M., Jackson A.P., Knoblich J.A. (2013). Cerebral organoids model human brain development and microcephaly. Nature.

[64] Muffat J., Li Y., Yuan B., Mitalipova M., Omer A., Corcoran S., Bakiasi G., Tsai L.H., Aubourg P., Ransohoff R.M. (2016). Efficient derivation of microglia-like cells from human pluripotent stem cells. Nat. Med..

[65] Raja W.K., Mungenast A.E., Lin Y.-T., Ko T., Abdurrob F., Seo J., Tsai L.-H. (2016). Self-Organizing 3D Human Neural Tissue Derived from Induced Pluripotent Stem Cells Recapitulate Alzheimer’s Disease Phenotypes. PLoS ONE.

[66] Ehrlich M., Hallmann A.L., Reinhardt P., Araúzo-Bravo M.J., Korr S., Röpke A., Psathaki O.E., Ehling P., Meuth S.G., Oblak A.L. (2015). Distinct Neurodegenerative Changes in an Induced Pluripotent Stem Cell Model of Frontotemporal Dementia Linked to Mutant TAU Protein. Stem Cell Rep..

[67] Sofroniew M.V., Vinters H.V. (2010). Astrocytes: Biology and pathology. Acta Neuropathol..

[68] Verkhratsky A., Ho M.S., Parpura V. (2019). Evolution of neuroglia. Advances in Experimental Medicine and Biology.

[69] Trujillo-Estrada L., Gomez-Arboledas A., Forner S., Martini A.C., Gutierrez A., Baglietto-Vargas D., LaFerla F.M. (2019). Astrocytes: From the Physiology to the Disease. Curr. Alzheimer Res..

[70] Chung W.S., Clarke L.E., Wang G.X., Stafford B.K., Sher A., Chakraborty C., Joung J., Foo L.C., Thompson A., Chen C. (2013). Astrocytes mediate synapse elimination through MEGF10 and MERTK pathways. Nature.

[71] Kim S.K., Nabekura J., Koizumi S. (2017). Astrocyte-mediated synapse remodeling in the pathological brain. Glia.

[72] Halassa M.M., Fellin T., Takano H., Dong J.H., Haydon P.G. (2007). Synaptic islands defined by the territory of a single astrocyte. J. Neurosci..

[73] Mahmoud S., Gharagozloo M., Simard C., Gris D. (2019). Astrocytes Maintain Glutamate Homeostasis in the CNS by Controlling the Balance between Glutamate Uptake and Release. Cells.

[74] Savtchouk I., Volterra A. (2018). Gliotransmission: Beyond black-and-white. J. Neurosci..

[75] Magistretti P.J., Allaman I. (2018). Lactate in the brain: From metabolic end-product to signalling molecule. Nat. Rev. Neurosci..

[76] Mächler P., Wyss M.T., Elsayed M., Stobart J., Gutierrez R., Von Faber-Castell A., Kaelin V., Zuend M., San Martín A., Romero-Gómez I. (2016). In Vivo Evidence for a Lactate Gradient from Astrocytes to Neurons. Cell Metab..

[77] Mishra A., Reynolds J.P., Chen Y., Gourine A.V., Rusakov D.A., Attwell D. (2016). Astrocytes mediate neurovascular signaling to capillary pericytes but not to arterioles. Nat. Neurosci..

[78] Sofroniew M.V. (2015). Astrocyte barriers to neurotoxic inflammation. Nat. Rev. Neurosci..

[79] Burda J.E., Bernstein A.M., Sofroniew M.V. (2016). Astrocyte roles in traumatic brain injury. Exp. Neurol..

[80] Schiweck J., Eickholt B.J., Murk K. (2018). Important shapeshifter: Mechanisms allowing astrocytes to respond to the changing nervous system during development, injury and disease. Front. Cell. Neurosci..

[81] Liddelow S.A., Guttenplan K.A., Clarke L.E., Bennett F.C., Bohlen C.J., Schirmer L., Bennett M.L., Münch A.E., Chung W.S., Peterson T.C. (2017). Neurotoxic reactive astrocytes are induced by activated microglia. Nature.

[82] Liddelow S.A., Barres B.A. (2017). Reactive Astrocytes: Production, Function, and Therapeutic Potential. Immunity.

[83] Khakh B.S., Deneen B. (2019). The Emerging Nature of Astrocyte Diversity. Annu. Rev. Neurosci..

[84] Itoh N., Itoh Y., Tassoni A., Ren E., Kaito M., Ohno A., Ao Y., Farkhondeh V., Johnsonbaugh H., Burda J. (2017). Cell-specific and region-specific transcriptomics in the multiple sclerosis model: Focus on astrocytes. Proc. Natl. Acad. Sci. USA.

[85] Diaz-Castro B., Gangwani M.R., Yu X., Coppola G., Khakh B.S. (2019). Astrocyte molecular signatures in Huntington’s disease. Sci. Transl. Med..

[86] Das S., Li Z., Noori A., Hyman B.T., Serrano-pozo A. (2020). Meta-analysis of mouse transcriptomic studies supports a context-dependent astrocyte reaction in acute CNS injury versus neurodegeneration. J. Neuroinflamm..

[87] Khakh B.S., Sofroniew M.V. (2015). Diversity of astrocyte functions and phenotypes in neural circuits. Nat. Neurosci..

[88] Ben Haim L., Rowitch D.H. (2016). Functional diversity of astrocytes in neural circuit regulation. Nat. Rev. Neurosci..

[89] Perez-Nievas B.G., Serrano-Pozo A. (2018). Deciphering the astrocyte reaction in Alzheimer’s disease. Front. Aging Neurosci..

[90] John Lin C.C., Yu K., Hatcher A., Huang T.W., Lee H.K., Carlson J., Weston M.C., Chen F., Zhang Y., Zhu W. (2017). Identification of diverse astrocyte populations and their malignant analogs. Nat. Neurosci..

[91] Batiuk M.Y., Martirosyan A., Wahis J., de Vin F., Marneffe C., Kusserow C., Koeppen J., Viana J.F., Oliveira J.F., Voet T. (2020). Identification of region-specific astrocyte subtypes at single cell resolution. Nat. Commun..

[92] Bayraktar O.A., Bartels T., Holmqvist S., Kleshchevnikov V., Martirosyan A., Polioudakis D., Ben Haim L., Young A.M.H., Batiuk M.Y., Prakash K. (2020). Astrocyte layers in the mammalian cerebral cortex revealed by a single-cell in situ transcriptomic map. Nat. Neurosci..

[93] Itagaki S., McGeer P.L., Akiyama H., Zhu S., Selkoe D. (1989). Relationship of microglia and astrocytes to amyloid deposits of Alzheimer disease. J. Neuroimmunol..

[94] Vehmas A.K., Kawas C.H., Stewart W.F., Troncoso J.C. (2003). Immune reactive cells in senile plaques and cognitive decline in Alzheimer’s disease. Neurobiol. Aging.

[95] Simpson J.E., Ince P.G., Shaw P.J., Heath P.R., Raman R., Garwood C.J., Gelsthorpe C., Baxter L., Forster G., Matthews F.E. (2011). Microarray analysis of the astrocyte transcriptome in the aging brain: Relationship to Alzheimer’s pathology and APOE genotype. Neurobiol. Aging.

[96] Olabarria M., Noristani H.N., Verkhratsky A., Rodríguez J.J. (2010). Concomitant astroglial atrophy and astrogliosis in a triple transgenic animal model of Alzheimer’s disease. Glia.

[97] Kraft A.W., Hu X., Yoon H., Yan P., Xiao Q., Wang Y., Gil S.C., Brown J., Wilhelmsson U., Restivo J.L. (2013). Attenuating astrocyte activation accelerates plaque pathogenesis in APP/PS1 mice. FASEB J..

[98] Xiao Q., Yan P., Ma X., Liu H., Perez R., Zhu A., Gonzales E., Burchett J.M., Schuler D.R., Cirrito J.R. (2014). Enhancing astrocytic lysosome biogenesis facilitates Aβ clearance and attenuates amyloid plaque pathogenesis. J. Neurosci..

[99] Gomez-Arboledas A., Davila J.C., Sanchez-Mejias E., Navarro V., Nuñez-Diaz C., Sanchez-Varo R., Sanchez-Mico M.V., Trujillo-Estrada L., Fernandez-Valenzuela J.J., Vizuete M. (2018). Phagocytic clearance of presynaptic dystrophies by reactive astrocytes in Alzheimer’s disease. Glia.

[100] Sekar S., McDonald J., Cuyugan L., Aldrich J., Kurdoglu A., Adkins J., Serrano G., Beach T.G., Craig D.W., Valla J. (2015). Alzheimer’s disease is associated with altered expression of genes involved in immune response and mitochondrial processes in astrocytes. Neurobiol. Aging.

[101] Orre M., Kamphuis W., Osborn L.M., Jansen A.H.P., Kooijman L., Bossers K., Hol E.M. (2014). Isolation of glia from Alzheimer’s mice reveals inflammation anddysfunction. Neurobiol. Aging.

[102] Zhou Y., Song W.M., Andhey P.S., Swain A., Levy T., Miller K.R., Poliani P.L., Cominelli M., Grover S., Gilfillan S. (2020). Human and mouse single-nucleus transcriptomics reveal TREM2-dependent and TREM2-independent cellular responses in Alzheimer’s disease. Nat. Med..

[103] Mathys H., Davila-Velderrain J., Peng Z., Gao F., Mohammadi S., Young J.Z., Menon M., He L., Abdurrob F., Jiang X. (2019). Single-cell transcriptomic analysis of Alzheimer’s disease. Nature.

[104] Grubman A., Chew G., Ouyang J.F., Sun G., Choo X.Y., McLean C., Simmons R.K., Buckberry S., Vargas-Landin D.B., Poppe D. (2019). A single-cell atlas of entorhinal cortex from individuals with Alzheimer’s disease reveals cell-type-specific gene expression regulation. Nat. Neurosci..

[105] Habib N., McCabe C., Medina S., Varshavsky M., Kitsberg D., Dvir-Szternfeld R., Green G., Dionne D., Nguyen L., Marshall J.L. (2020). Disease-associated astrocytes in Alzheimer’s disease and aging. Nat. Neurosci..

[106] Keren-Shaul H., Spinrad A., Weiner A., Matcovitch-Natan O., Dvir-Szternfeld R., Ulland T.K., David E., Baruch K., Lara-Astaiso D., Toth B. (2017). A Unique Microglia Type Associated with Restricting Development of Alzheimer’s Disease. Cell.

[107] Krasemann S., Madore C., Cialic R., Baufeld C., Calcagno N., El Fatimy R., Beckers L., O’Loughlin E., Xu Y., Fanek Z. (2017). The TREM2-APOE Pathway Drives the Transcriptional Phenotype of Dysfunctional Microglia in Neurodegenerative Diseases. Immunity.

[108] Chen W.-T., Lu A., Craessaerts K., Pavie B., Sala Frigerio C., Corthout N., Qian X., Laláková J., Kühnemund M., Voytyuk I. (2020). Spatial Transcriptomics and In Situ Sequencing to Study Alzheimer’s Disease. Cell.

[109] Vasile F., Dossi E., Rouach N. (2017). Human astrocytes: Structure and functions in the healthy brain. Brain Struct. Funct..

[110] Oberheim N.A., Takano T., Han X., He W., Lin J.H.C., Wang F., Xu Q., Wyatt J.D., Pilcher W., Ojemann J.G. (2009). Uniquely hominid features of adult human astrocytes. J. Neurosci..

[111] Zhang Y., Sloan S.A., Clarke L.E., Caneda C., Plaza C.A., Blumenthal P.D., Vogel H., Steinberg G.K., Edwards M.S.B., Li G. (2016). Purification and Characterization of Progenitor and Mature Human Astrocytes Reveals Transcriptional and Functional Differences with Mouse. Neuron.

[112] Krencik R., Weick J.P., Liu Y., Zhang Z.-J., Zhang S.-C. (2011). Specification of transplantable astroglial subtypes from human pluripotent stem cells. Nat. Biotechnol..

[113] Shaltouki A., Peng J., Liu Q., Rao M.S., Zeng X. (2013). Efficient generation of astrocytes from human pluripotent stem cells in defined conditions. Stem Cells.

[114] Santos R., Vadodaria K.C., Jaeger B.N., Mei A., Lefcochilos-Fogelquist S., Mendes A.P.D., Erikson G., Shokhirev M., Randolph-Moore L., Fredlender C. (2017). Differentiation of Inflammation-Responsive Astrocytes from Glial Progenitors Generated from Human Induced Pluripotent Stem Cells. Stem Cell Rep..

[115] TCW J., Wang M., Pimenova A.A., Bowles K.R., Hartley B.J., Lacin E., Machlovi S.I., Abdelaal R., Karch C.M., Phatnani H. (2017). An Efficient Platform for Astrocyte Differentiation from Human Induced Pluripotent Stem Cells. Stem Cell Rep..

[116] Chambers C.B., Peng Y., Nguyen H., Gaiano N., Fishell G., Nye J.S. (2001). Spatiotemporal selectivity of response to Notch1 signals in mammalian forebrain precursors. Development.

[117] Tsai H.H., Li H., Fuentealba L.C., Molofsky A.V., Taveira-Marques R., Zhuang H., Tenney A., Murnen A.T., Fancy S.P.J., Merkle F. (2012). Regional astrocyte allocation regulates CNS synaptogenesis and repair. Science.

[118] Perriot S., Mathias A., Perriard G., Canales M., Jonkmans N., Merienne N., Meunier C., El Kassar L., Perrier A.L., Laplaud D.A. (2018). Human Induced Pluripotent Stem Cell-Derived Astrocytes Are Differentially Activated by Multiple Sclerosis-Associated Cytokines. Stem Cell Rep..

[119] Canals I., Ginisty A., Quist E., Timmerman R., Fritze J., Miskinyte G., Monni E., Hansen M.G., Hidalgo I., Bryder D. (2018). Rapid and efficient induction of functional astrocytes from human pluripotent stem cells. Nat. Methods.

[120] Li X., Tao Y., Bradley R., Du Z., Tao Y., Kong L., Dong Y., Jones J., Yan Y., Harder C.R.K. (2018). Fast Generation of Functional Subtype Astrocytes from Human Pluripotent Stem Cells. Stem Cell Rep..

[121] Tchieu J., Calder E.L., Guttikonda S.R., Gutzwiller E.M., Aromolaran K.A., Steinbeck J.A., Goldstein P.A., Studer L. (2019). NFIA is a gliogenic switch enabling rapid derivation of functional human astrocytes from pluripotent stem cells. Nat. Biotechnol..

[122] Sloan S.A., Darmanis S., Huber N., Khan T.A., Birey F., Caneda C., Reimer R., Quake S.R., Barres B.A., Paşca S.P. (2017). Human Astrocyte Maturation Captured in 3D Cerebral Cortical Spheroids Derived from Pluripotent Stem Cells. Neuron.

[123] Kondo T., Asai M., Tsukita K., Kutoku Y., Ohsawa Y., Sunada Y., Imamura K., Egawa N., Yahata N., Okita K. (2013). Modeling Alzheimer’s Disease with iPSCs Reveals Stress Phenotypes Associated with Intracellular Aβ and Differential Drug Responsiveness. Cell Stem Cell.

[124] Oksanen M., Petersen A.J., Naumenko N., Puttonen K., Lehtonen Š., Gubert Olivé M., Shakirzyanova A., Leskelä S., Sarajärvi T., Viitanen M. (2017). PSEN1 Mutant iPSC-Derived Model Reveals Severe Astrocyte Pathology in Alzheimer’s Disease. Stem Cell Rep..

[125] Jones V.C., Atkinson-Dell R., Verkhratsky A., Mohamet L. (2017). Aberrant iPSC-derived human astrocytes in Alzheimer’s disease. Cell Death Dis..

[126] Zhao J., Davis M.D., Martens Y.A., Shinohara M., Graff-Radford N.R., Younkin S.G., Wszolek Z.K., Kanekiyo T., Bu G. (2017). APOE ε4/ε4 diminishes neurotrophic function of human iPSC-derived astrocytes. Hum. Mol. Genet..

[127] Hallmann A.-L., Araúzo-Bravo M.J., Mavrommatis L., Ehrlich M., Röpke A., Brockhaus J., Missler M., Sterneckert J., Schöler H.R., Kuhlmann T. (2017). Astrocyte pathology in a human neural stem cell model of frontotemporal dementia caused by mutant TAU protein. Sci. Rep..

[128] Franklin R.J.M., Ffrench-Constant C., Edgar J.M., Smith K.J. (2012). Neuroprotection and repair in multiple sclerosis. Nat. Rev. Neurol..

[129] Li L., Tian E., Chen X., Chao J., Klein J., Qu Q., Sun G., Sun G., Huang Y., Warden C.D. (2018). GFAP Mutations in Astrocytes Impair Oligodendrocyte Progenitor Proliferation and Myelination in an hiPSC Model of Alexander Disease. Cell Stem Cell.

[130] Lee Y., Morrison B.M., Li Y., Lengacher S., Farah M.H., Hoffman P.N., Liu Y., Tsingalia A., Jin L., Zhang P.-W. (2012). Oligodendroglia metabolically support axons and contribute to neurodegeneration. Nature.

[131] Osipovitch M., Asenjo Martinez A., Mariani J.N., Cornwell A., Dhaliwal S., Zou L., Chandler-Militello D., Wang S., Li X., Benraiss S.J. (2019). Human ESC-Derived Chimeric Mouse Models of Huntington’s Disease Reveal Cell-Intrinsic Defects in Glial Progenitor Cell Differentiation. Cell Stem Cell.

[132] Windrem M.S., Osipovitch M., Liu Z., Bates J., Chandler-Militello D., Zou L., Munir J., Schanz S., McCoy K., Miller R.H. (2017). Human iPSC Glial Mouse Chimeras Reveal Glial Contributions to Schizophrenia. Cell Stem Cell.

[133] de Vrij F.M., Bouwkamp C.G., Gunhanlar N., Shpak G., Lendemeijer B., Baghdadi M., Gopalakrishna S., Ghazvini M., Li T.M., Quadri M. (2019). Candidate CSPG4 mutations and induced pluripotent stem cell modeling implicate oligodendrocyte progenitor cell dysfunction in familial schizophrenia. Mol. Psychiatr..

[134] Nasrabady S.E., Rizvi B., Goldman J.E., Brickman A.M. (2018). White matter changes in Alzheimer’s disease: A focus on myelin and oligodendrocytes. Acta Neuropathol. Commun..

[135] Li L., Li R., Zacharek A., Wang F., Landschoot-ward J. (2020). ABCA1/ApoE/HDL Signaling Pathway Facilitates Myelination and Oligodendrogenesis after Stroke. Int. J. Mol. Sci..

[136] Desai M.K., Mastrangelo M.A., Ryan D.A., Sudol K.L., Narrow W.C., Bowers W.J. (2010). Early oligodendrocyte/myelin pathology in Alzheimer’s disease mice constitutes a novel therapeutic target. Am. J. Pathol..

[137] Falcão A.M., van Bruggen D., Marques S., Meijer M., Jäkel S., Agirre E., Samudyata, Floriddia E.M., Vanichkina D.P., French-Constant C. (2018). Disease-specific oligodendrocyte lineage cells arise in multiple sclerosis. Nat. Med..

[138] Chanoumidou K., Mozafari S., Baron-Van Evercooren A., Kuhlmann T. (2019). Stem cell derived oligodendrocytes to study myelin diseases. Glia.

[139] Wang S., Bates J., Li X., Schanz S., Chandler-Militello D., Levine C., Maherali N., Studer L., Hochedlinger K., Windrem M. (2013). Human iPSC-derived oligodendrocyte progenitor cells can myelinate and rescue a mouse model of congenital hypomyelination. Cell Stem Cell.

[140] Douvaras P., Wang J., Zimmer M., Hanchuk S., O’Bara M.A., Sadiq S., Sim F.J., Goldman J., Fossati V. (2014). Efficient generation of myelinating oligodendrocytes from primary progressive multiple sclerosis patients by induced pluripotent stem cells. Stem Cell Rep..

[141] García-León J.A., Kumar M., Boon R., Chau D., One J., Wolfs E., Eggermont K., Berckmans P., Gunhanlar N., de Vrij F. (2018). SOX10 Single Transcription Factor-Based Fast and Efficient Generation of Oligodendrocytes from Human Pluripotent Stem Cells. Stem Cell Rep..

[142] Ehrlich M., Mozafari S., Glatza M., Starost L., Velychko S., Hallmann A.-L., Cui Q.-L., Schambach A., Kim K.-P., Bachelin C. (2017). Rapid and efficient generation of oligodendrocytes from human induced pluripotent stem cells using transcription factors. Proc. Natl. Acad. Sci. USA.

[143] Colonna M., Butovsky O. (2017). Microglia Function in the Central Nervous System During Health and Neurodegeneration. Annu. Rev. Immunol..

[144] Hickman S., Izzy S., Sen P., Morsett L., El Khoury J. (2018). Microglia in neurodegeneration. Nat. Neurosci..

[145] Thion M.S., Ginhoux F., Garel S. (2018). Microglia and early brain development: An intimate journey. Science.

[146] Prinz M., Jung S., Priller J. (2019). Microglia Biology: One Century of Evolving Concepts. Cell.

[147] Madore C., Yin Z., Leibowitz J., Butovsky O. (2020). Microglia, Lifestyle Stress, and Neurodegeneration. Immunity.

[148] Ginhoux F., Greter M., Leboeuf M., Nandi S., See P., Gokhan S., Mehler M.F., Conway S.J., Ng L.G., Stanley E.R. (2010). Primitive Macrophages. Science.

[149] Kierdorf K., Erny D., Goldmann T., Sander V., Schulz C., Perdiguero E.G., Wieghofer P., Heinrich A., Riemke P., Hölscher C. (2013). Microglia emerge from erythromyeloid precursors via Pu.1-and Irf8-dependent pathways. Nat. Neurosci..

[150] Sierra A., Paolicelli R.C., Kettenmann H. (2019). Cien Años de Microglía: Milestones in a Century of Microglial Research. Trends Neurosci..

[151] Tay T.L., Mai D., Dautzenberg J., Fernández-Klett F., Lin G., Sagar, Datta M., Drougard A., Stempfl T., Ardura-Fabregat A. (2017). A new fate mapping system reveals context-dependent random or clonal expansion of microglia. Nat. Neurosci..

[152] Huang Y., Xu Z., Xiong S., Sun F., Qin G., Hu G., Wang J., Zhao L., Liang Y.X., Wu T. (2018). Repopulated microglia are solely derived from the proliferation of residual microglia after acute depletion. Nat. Neurosci..

[153] Tan Y.L., Yuan Y., Tian L. (2020). Microglial regional heterogeneity and its role in the brain. Mol. Psychiatr..

[154] Sanchez-Mejias E., Navarro V., Jimenez S., Sanchez-Mico M., Sanchez-Varo R., Nuñez-Diaz C., Trujillo-Estrada L., Davila J.C., Vizuete M., Gutierrez A. (2016). Soluble phospho-tau from Alzheimer’s disease hippocampus drives microglial degeneration. Acta Neuropathol..

[155] Zhang B., Gaiteri C., Bodea L.-G., Wang Z., McElwee J., Podtelezhnikov A.A., Zhang C., Xie T., Tran L., Dobrin R. (2013). Integrated systems approach identifies genetic nodes and networks in late-onset Alzheimer’s disease. Cell.

[156] Gratuze M., Leyns C.E.G., Holtzman D.M. (2018). New insights into the role of TREM2 in Alzheimer’s disease. Mol. Neurodegener..

[157] Cheng-Hathaway P.J., Reed-Geaghan E.G., Jay T.R., Casali B.T., Bemiller S.M., Puntambekar S.S., von Saucken V.E., Williams R.Y., Karlo J.C., Moutinho M. (2018). The Trem2 R47H variant confers loss-of-function-like phenotypes in Alzheimer’s disease. Mol. Neurodegener..

[158] Xiang X., Piers T.M., Wefers B., Zhu K., Mallach A., Brunner B., Kleinberger G., Song W., Colonna M., Herms J. (2018). The Trem2 R47H Alzheimer’s risk variant impairs splicing and reduces Trem2 mRNA and protein in mice but not in humans. Mol. Neurodegener..

[159] Galatro T.F., Holtman I.R., Lerario A.M., Vainchtein I.D., Brouwer N., Sola P.R., Veras M.M., Pereira T.F., Leite R.E.P., Möller T. (2017). Transcriptomic analysis of purified human cortical microglia reveals age-associated changes. Nat. Neurosci..

[160] Ohgidani M., Kato T.A., Setoyama D., Sagata N., Hashimoto R., Shigenobu K., Yoshida T., Hayakawa K., Shimokawa N., Miura D. (2014). Direct induction of ramified microglia-like cells from human monocytes: Dynamic microglial dysfunction in Nasu-Hakola disease. Sci. Rep..

[161] Beutner C., Roy K., Linnartz B., Napoli I., Neumann H. (2010). Generation of microglial cells from mouse embryonic stem cells. Nat. Protoc..

[162] Pandya H., Shen M.J., Ichikawa D.M., Sedlock A.B., Choi Y., Johnson K.R., Kim G., Brown M.A., Elkahloun A.G., Maric D. (2017). Differentiation of human and murine induced pluripotent stem cells to microglia-like cells. Nat. Neurosci..

[163] Abud E.M., Ramirez R.N., Martinez E.S., Healy L.M., Nguyen C.H.H., Newman S.A., Yeromin A.V., Scarfone V.M., Marsh S.E., Fimbres C. (2017). iPSC-Derived Human Microglia-like Cells to Study Neurological Diseases. Neuron.

[164] Douvaras P., Sun B., Wang M., Kruglikov I., Lallos G., Zimmer M., Terrenoire C., Zhang B., Gandy S., Schadt E. (2017). Directed Differentiation of Human Pluripotent Stem Cells to Microglia. Stem Cell Rep..

[165] Haenseler W., Sansom S.N., Buchrieser J., Newey S.E., Moore C.S., Nicholls F.J., Chintawar S., Schnell C., Antel J.P., Allen N.D. (2017). A Highly Efficient Human Pluripotent Stem Cell Microglia Model Displays a Neuronal-Co-culture-Specific Expression Profile and Inflammatory Response. Stem Cell Rep..

[166] Takata K., Kozaki T., Lee C.Z.W., Thion M.S., Otsuka M., Lim S., Utami K.H., Fidan K., Park D.S., Malleret B. (2017). Induced-Pluripotent-Stem-Cell-Derived Primitive Macrophages Provide a Platform for Modeling Tissue-Resident Macrophage Differentiation and Function. Immunity.

[167] McQuade A., Coburn M., Tu C.H., Hasselmann J., Davtyan H., Blurton-Jones M. (2018). Development and validation of a simplified method to generate human microglia from pluripotent stem cells. Mol. Neurodegener..

[168] Butovsky O., Jedrychowski M.P., Moore C.S., Cialic R., Lanser A.J., Gabriely G., Koeglsperger T., Dake B., Wu P.M., Doykan C.E. (2014). Identification of a unique TGF-β–dependent molecular and functional signature in microglia. Nat. Neurosci..

[169] Espuny-Camacho I., Arranz A.M., Fiers M., Snellinx A., Ando K., Munck S., Bonnefont J., Lambot L., Corthout N., Omodho L. (2017). Hallmarks of Alzheimer’s Disease in Stem-Cell-Derived Human Neurons Transplanted into Mouse Brain. Neuron.

[170] Hasselmann J., Coburn M.A., England W., Figueroa Velez D.X., Kiani Shabestari S., Tu C.H., McQuade A., Kolahdouzan M., Echeverria K., Claes C. (2019). Development of a Chimeric Model to Study and Manipulate Human Microglia In Vivo. Neuron.

[171] Svoboda D.S., Barrasa M.I., Shu J., Rietjens R., Zhang S., Mitalipova M., Berube P., Fu D., Shultz L.D., Bell G.W. (2019). Human iPSC-derived microglia assume a primary microglia-like state after transplantation into the neonatal mouse brain. Proc. Natl. Acad. Sci. USA.

[172] Xu R., Li X., Boreland A.J., Posyton A., Kwan K., Hart R.P., Jiang P. (2020). Human iPSC-derived mature microglia retain their identity and functionally integrate in the chimeric mouse brain. Nat. Commun..

[173] Mancuso R., Van Den Daele J., Fattorelli N., Wolfs L., Balusu S., Burton O., Liston A., Sierksma A., Fourne Y., Poovathingal S. (2019). Stem-cell-derived human microglia transplanted in mouse brain to study human disease. Nat. Neurosci..

[174] Brownjohn P.W., Smith J., Solanki R., Lohmann E., Houlden H., Hardy J., Dietmann S., Livesey F.J. (2018). Functional Studies of Missense TREM2 Mutations in Human Stem Cell-Derived Microglia. Stem Cell Rep..

[175] Garcia-Reitboeck P., Phillips A., Piers T.M., Villegas-Llerena C., Butler M., Mallach A., Rodrigues C., Arber C.E., Heslegrave A., Zetterberg H. (2018). Human Induced Pluripotent Stem Cell-Derived Microglia-Like Cells Harboring TREM2 Missense Mutations Show Specific Deficits in Phagocytosis. Cell Rep..

[176] Claes C., Van Den Daele J., Boon R., Schouteden S., Colombo A., Monasor L.S., Fiers M., Ordovás L., Nami F., Bohrmann B. (2018). Human stem cell–derived monocytes and microglia-like cells reveal impaired amyloid plaque clearance upon heterozygous or homozygous loss of TREM2. Alzheimer’s Dement..

[177] Piers T.M., Cosker K., Mallach A., Johnson G.T., Guerreiro R., Hardy J., Pocock J.M. (2020). A locked immunometabolic switch underlies TREM2 R47H loss of function in human iPSC-derived microglia. FASEB J..

[178] Konttinen H., Cabral-da-Silva M.E.C., Ohtonen S., Wojciechowski S., Shakirzyanova A., Caligola S., Giugno R., Ishchenko Y., Hernández D., Fazaludeen M.F. (2019). PSEN1ΔE9, APPswe, and APOE4 Confer Disparate Phenotypes in Human iPSC-Derived Microglia. Stem Cell Rep..

[179] Hansen E., Krautwald M., MacZurek A.E., Stuchbury G., Fromm P., Steele M., Schulz O., Garcia O.B., Castillo J., Körner H. (2010). A versatile high throughput screening system for the simultaneous identification of anti-inflammatory and neuroprotective compounds. J. Alzheimer’s Dis..

[180] Figuera-Losada M., Thomas A.G., Stathis M., Stockwell B.R., Rojas C., Slusher B.S. (2017). Development of a primary microglia screening assay and its use to characterize inhibition of system xc- by erastin and its analogs. Biochem. Biophys. Rep..

[181] Rustenhoven J., Smith A.M., Smyth L.C., Jansson D., Scotter E.L., Swanson M.E.V., Aalderink M., Coppieters N., Narayan P., Handley R. (2018). 1 regulates Alzheimer’s disease-associated genes in primary human microglia. Mol. Neurodegener..

[182] Cakir B., Xiang Y., Tanaka Y., Kural M.H., Parent M., Kang Y.J., Chapeton K., Patterson B., Yuan Y., He C.S. (2019). Engineering of human brain organoids with a functional vascular-like system. Nat. Methods.

[183] Ormel P.R., Vieira de Sá R., van Bodegraven E.J., Karst H., Harschnitz O., Sneeboer M.A.M., Johansen L.E., van Dijk R.E., Scheefhals N., Berdenis van Berlekom A. (2018). Microglia innately develop within cerebral organoids. Nat. Commun..

[184] Pellegrini L., Bonfio C., Chadwick J., Begum F., Skehel M., Lancaster M.A. (2020). Human CNS barrier-forming organoids with cerebrospinal fluid production. Science.

[185] Choi S.H., Kim Y.H., Hebisch M., Sliwinski C., Lee S., D’Avanzo C., Chen H., Hooli B., Asselin C., Muffat J. (2014). A three-dimensional human neural cell culture model of Alzheimer’s disease. Nature.

[186] Jorfi M., D’Avanzo C., Tanzi R.E., Kim D.Y., Irimia D. (2018). Human Neurospheroid Arrays for In Vitro Studies of Alzheimer’s Disease. Sci. Rep..

[187] Gonzalez C., Armijo E., Bravo-Alegria J., Becerra-Calixto A., Mays C.E., Soto C. (2018). Modeling amyloid beta and tau pathology in human cerebral organoids. Mol. Psychiatr..

[188] Zhang D., Pekkanen-Mattila M., Shahsavani M., Falk A., Teixeira A.I., Herland A. (2014). A 3D Alzheimer’s disease culture model and the induction of P21-activated kinase mediated sensing in iPSC derived neurons. Biomaterials.

[189] Labour M.N., Vigier S., Lerner D., Marcilhac A., Belamie E. (2016). 3D compartmented model to study the neurite-related toxicity of Aβ aggregates included in collagen gels of adaptable porosity. Acta Biomater..

[190] Simpson L.W., Szeto G.L., Boukari H., Good T.A., Leach J.B. (2020). Collagen hydrogel confinement of Amyloid-β (Aβ) accelerates aggregation and reduces cytotoxic effects. Acta Biomater..

[191] Park J., Wetzel I., Marriott I., Dréau D., D’Avanzo C., Kim D.Y., Tanzi R.E., Cho H. (2018). A 3D human triculture system modeling neurodegeneration and neuroinflammation in Alzheimer’s disease. Nat. Neurosci..

[192] Kwak S.S., Washicosky K.J., Brand E., von Maydell D., Aronson J., Kim S., Capen D.E., Cetinbas M., Sadreyev R., Ning S. (2020). Amyloid-β42/40 ratio drives tau pathology in 3D human neural cell culture models of Alzheimer’s disease. Nat. Commun..

[193] Choi H., Kim H.J., Yang J., Chae S., Lee W., Chung S., Kim J., Choi H., Song H., Lee C.K. (2020). Acetylation changes tau interactome to degrade tau in Alzheimer’s disease animal and organoid models. Aging Cell.

[194] Mueller S.G., Schuff N., Yaffe K., Madison C., Miller B., Weiner M.W. (2010). Hippocampal atrophy patterns in mild cognitive impairment and alzheimer’s disease. Hum. Brain Mapp..

[195] Pomeshchik Y., Klementieva O., Gil J., Martinsson I., Hansen M.G., de Vries T., Sancho-Balsells A., Russ K., Savchenko E., Collin A. (2020). Human iPSC-Derived Hippocampal Spheroids: An Innovative Tool for Stratifying Alzheimer Disease Patient-Specific Cellular Phenotypes and Developing Therapies. Stem Cell Rep..

